# PROTAC Technology as a New Tool for Modern Pharmacotherapy

**DOI:** 10.3390/molecules30102123

**Published:** 2025-05-11

**Authors:** Natalia Kubryń, Łukasz Fijałkowski, Jacek Nowaczyk, Amer Jamil, Alicja Nowaczyk

**Affiliations:** 1 Department of Organic Chemistry, Faculty of Pharmacy, Ludwik Rydygier Collegium Medicum in Bydgoszcz, Nicolaus Copernicus University in Toruń, 2 dr. A. Jurasza St., 85-094 Bydgoszcz, Poland; natalia.kubryn@doktorant.umk.pl (N.K.); alicja@cm.umk.pl (A.N.); 2 Department of Physical Chemistry and Physicochemistry of Polymers, Faculty of Chemistry, Nicolaus Copernicus University, 7 Gagarina St., 87-100 Toruń, Poland; jacek.nowaczyk@umk.pl; 3 Department of Biochemistry, University of Agriculture, Faisalabad 38040, Pakistan; amerjamil@uaf.edu.pk

**Keywords:** PROTACs, degradation, ligands, cancer

## Abstract

The publication focuses on the innovative applications of PROTAC (proteolysis-targeting chimera) technology in modern pharmacotherapy, with particular emphasis on cancer treatment. PROTACs represent an advanced therapeutic strategy that enables selective protein degradation, opening new possibilities in drug design. This technology shows potential in the treatment of cancers, viral infections (such as HIV and COVID-19), and chronic diseases including atherosclerosis, Alzheimer’s disease, atopic dermatitis, and Huntington’s disease. Promising results from clinical studies on the compound ARV-471 confirm the effectiveness of this approach. New types of PROTACs, like TF-PROTAC and PhosphoTAC, are designed to enhance the effectiveness, stability, and absorption of treatment drugs. The conclusions of the review highlight the broad therapeutic potential of PROTACs in various diseases and their relevance for the future of therapies, particularly in oncology.

## 1. Introduction

Cancer is one of the leading causes of death worldwide, resulting in significant health and economic losses. In 2022, nearly 20 million new cancer cases were diagnosed, and the number of cancer-related deaths reached 9.7 million. Estimates suggest that approximately one in five people, regardless of gender, will develop cancer during their lifetime, while around one in nine men and one in twelve women die from the disease [1,2]. Despite substantial advances in the diagnosis and treatment of many types of cancer, challenges remain regarding effectiveness, toxicity, and resistance to treatment, necessitating innovative therapeutic solutions. Traditional treatment methods, such as surgery, radiotherapy, and chemotherapy, have evolved over decades, but they are burdened with numerous limitations, including insufficient selectivity towards cancer cells and severe side effects [3,4]. In recent years, new promising strategies have emerged in the field of anticancer therapy, such as immunotherapy, targeted therapies, and, particularly interesting, the technology of targeted protein degradation known as PROTACs (proteolysis-targeting chimeras) [5]. Understanding the mechanisms of action and potential clinical applications of PROTACs could lead to breakthroughs in the treatment of various types of cancer, opening up new therapeutic possibilities that are more effective and less toxic for patients [6].

PROTACs use a strategy that harnesses the ubiquitin–proteasome system to target specific proteins and induce their degradation within the cell. The normal physiological function of the ubiquitin–proteasome system is to clear denatured, mutated, or harmful proteins from cells [7]. PROTACs exploit this protein destruction mechanism to specifically remove targeted proteins. To date, PROTAC technology can be used to target a variety of proteins, including transcription factors, structural proteins, enzymes, and regulatory proteins [8]. Recently, this technology has attracted significant attention from researchers across various fields, from cancer to neurodegenerative diseases. This interest is primarily due to the potent ability of designed PROTAC molecules to induce targeted protein degradation. Numerous studies have shown that degrading a protein can be more effective than merely inhibiting it for anticancer activities [9,10,11].

**Figure 1:**
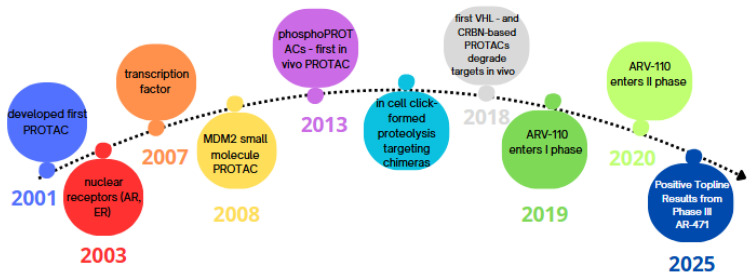
History of PROTACs.

More than two decades after Craig M. Crews developed the first PROTAC in 2001, this technology has achieved a significant breakthrough. A short history of the development of PROTACs is presented in Figure 1. Until now, PROTACs were large molecules that were not absorbed through the gastrointestinal tract, limiting their application. A groundbreaking achievement was the development of the first orally bioavailable small-molecule PROTAC-ARV-110. It was designed and synthesised to degrade the androgen receptor (AR) and is used in the treatment of metastatic castration-resistant prostate cancer [12].

## 2. PROTAC Design

### 2.1. Protein of Interest (POI)

Initial PROTACs that entered clinical phases were aimed at classic targets that have already been clinically validated, allowing for the assessment of safety and efficacy in vivo, thus reinforcing the position of PROTACs as an effective tool for the treatment of solid tumours. However, the truly exciting possibility offered by PROTAC technology is its ability to target “hard-to-treat” or “undruggable” proteins [13]. This strategy differs significantly from traditional small molecule inhibitors, opening up new therapeutic avenues. Currently, there is no “gold standard” for PROTAC targets, akin to Lipinski’s Rule of Five. An ideal target for PROTACs should meet several of the following criteria:

1. Pathogenic alterations: the target should exhibit pathogenic gain-of-function alterations, such as overexpression, mutations, or changes in localisation [14].
2. Ligand-binding pocket: it should possess a site where the PROTAC molecule can attach [15].
3. Ubiquitination site: there should be a location on the target’s surface that allows for the binding of the E3 ubiquitin ligase [16].
4. Structure: the target should have a flexible structure that can be processed by the proteasome [17].

The PROTACs currently being studied have shown their effectiveness in binding to the POI in numerous in vitro and in vivo studies. High affinity or covalent binding between the POI and the PROTAC (often referred to as warhead) is not a requisite for efficacy. Research on the multi-kinase inhibitor, foretinib, shows that PROTACs with this inhibitor only broke down a small amount of the related kinases. The overall success of kinase degradation relied more on how well the POI–PROTAC–E3 ubiquitin ligase complex was formed, rather than how strongly foretinib attached to the target protein. High affinity or covalent binding between the POI and the warhead might make it harder for the PROTACs to detach from the ternary complex, which is important for ongoing protein breakdown [18]. Excessive binding could lead to an occupancy-driven mechanism rather than an event-driven one, limiting the benefits of using PROTACs. PROTAC technology offers an innovative approach to therapy, enabling the targeting of difficult-to-treat POI proteins. Ongoing research into PROTACs continues to optimise targets and warheads, which may lead to new, effective therapies. Although there is no perfect method for choosing PROTAC targets yet, studying how well ternary complexes form and how to pick the right ligands gives us hope for solving current problems with targeting difficult proteins [19].

### 2.2. Linker

The main challenge in creating PROTAC linkers is choosing the right spots on the warhead and the E3 ubiquitin ligase ligand. The ideal linker attachment point should allow it to access the ligand-binding pocket or a solvent-accessible region while avoiding interference with the binding between the small molecule and its POI [20,21]. Considering availability and reaction efficiency, researchers typically seek linker points from active ligand atoms, such as carboxyl and amine groups [22,23]. In the case of larger ligands, the linker may be attached while simultaneously removing some non-essential groups, which does not affect the targeting of the POI or the recruitment of the E3 ubiquitin ligase [24,25]. For small molecules with multiple potential linker points, it is essential to consider whether groups in the solvent-exposed region are also required for binding between the molecule and the protein, which often necessitates experimental validation. There is no single design rule for linkers that guarantees the successful breakdown of target proteins, but altering the structure or length of the linker can greatly change how the whole PROTAC structure behaves (Table 1).

### 2.3. E3 Ligase

Ohoka et al. developed a PROTAC based on the AhR E3 ligase for the degradation of CRABP1, and Li et al. described the development of a PROTAC with the DCAF15 E3 ligase targeting BRD4 [26]. Approaches based on covalent bindings have become an attractive option for PROTAC development. For instance, Ward et al. developed a PROTAC targeting BRD4 by recruiting the RNF4 E3 ligase, while Nomura et al. directed the FEM1B E3 ligase for the degradation of BRD4 and BCR-ABL [27,28]. Single-cell RNA sequencing (scRNA-seq) techniques allow the study of gene expression profiles in individual cells, enabling the identification of the expression patterns of E3 ligases specific to cell types, which may have therapeutic implications. Various predictive models and databases assist in identifying E3 ligases and their targets. Medvar et al. created a database of 377 human E3 ubiquitin ligases, employing Bayesian techniques for the probabilistic ranking of ligases [29]. Park et al. proposed the CKSAAP model for predicting E3 ligase-target interactions [30]. Palomba et al. introduced the ELIOT platform, which contains information about E3 ligase pockets, aiding in PROTAC design [30,31].

**Figure 2:**
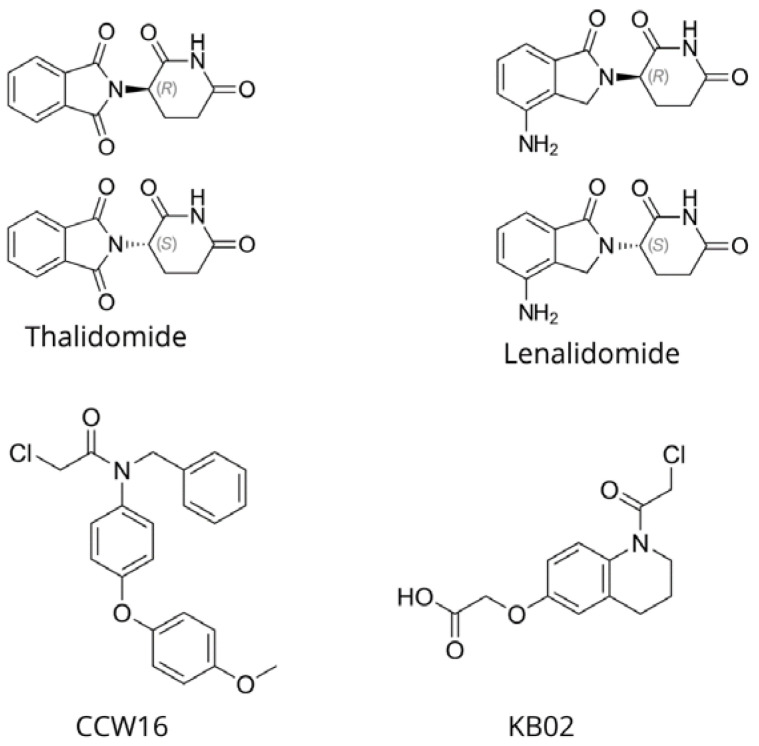
Selected E3 ligands.

Despite advancements, it is still hard to find good E3 ligase ligands, which restricts how these ligases can be used in PROTAC. In the past, researchers have used ligases like MDM2 and cIAP1, but they faced limitations due to their degradation efficiency and toxicity. Researchers focus on E3 ligases highly expressed in tumours, which may enhance the tissue-specific and anti-cancer activity of the PROTAC while minimising toxicity to other tissues. In conclusion, E3 ligases play a crucial role in protein degradation and hold immense therapeutic potential. Their utilisation in PROTAC technology could lead to the development of new, effective therapies for various diseases, especially cancers. Bringing together genomic and proteomic information, along with advanced prediction methods, could help us understand and use E3 ligases better in medicine [32,33,34]. Figure 2 shows examples of selected E3 ligands most commonly used in PROTAC technology.

## 3. Mechanism of Action

Traditional inhibitor compounds work by attaching to the binding pocket or active site of the target protein, which leads to a loss of function. Table 2 presents a comparison of PROTAC with traditional inhibitors [35,36].

**Figure 3:**
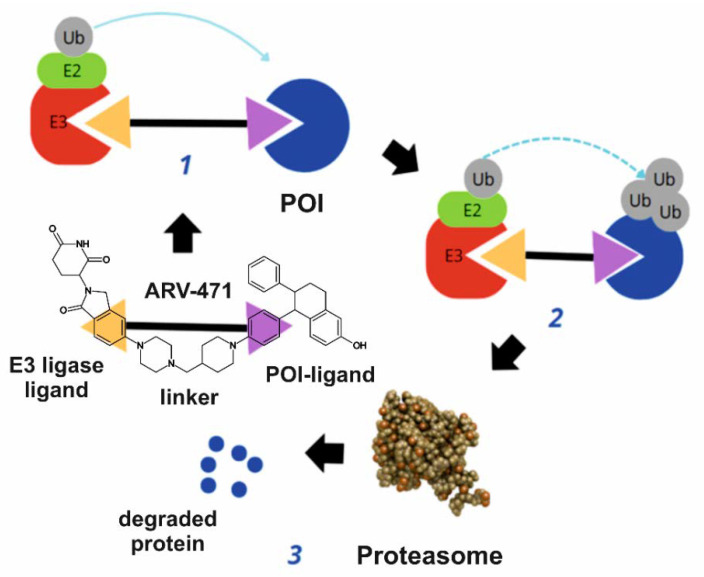
General mechanism of action of PROTAC. The ARV-471 is presented as an example molecule. **1**. First, E1 activates and conjugates ubiquitin with E2. Then, E2 forms a complex with the E3 ligase. **2**. The E3 ligase targets specific proteins and covalently attaches ubiquitin to the protein of interest. **3**. Finally, after the formation of a ubiquitin chain, the protein is recognised and degraded by the 26S proteasome. Ub: Ubiquitin, E1, E2, E3: ubiquitination enzymes.

Most enzyme inhibitors attach to proteins without forming strong bonds and can easily come off, so they need to be given in higher amounts to keep the active site occupied and remain effective in treatment. In contrast, PROTACs function through an event-driven mechanism. PROTACs need to bind to their target only long enough to facilitate the recruitment of the E3 ligase and the POI, leading to the degradation of the POI [37]. Essentially, they only need to interact briefly to induce the proteolysis of the POI by the proteasome. Once the target protein is degraded, PROTACs detach and can be reused to target additional proteins. This allows the PROTACs to remain active through multiple cycles of target degradation. Figure 3 illustrates the mechanism of action of PROTACs, highlighting their ability to induce targeted protein degradation. This way of working means that high amounts of the drug are not needed, allowing the use of lower doses that helps avoid many problems linked to traditional drugs. This characteristic not only reduces the required dosage but also minimises potential side effects, enhancing the therapeutic profile of PROTACs. Additionally, the ability of PROTACs to be recycled for multiple uses further increases their efficiency and therapeutic potential, making them a promising avenue for cancer treatment [38].

**Figure 4:**
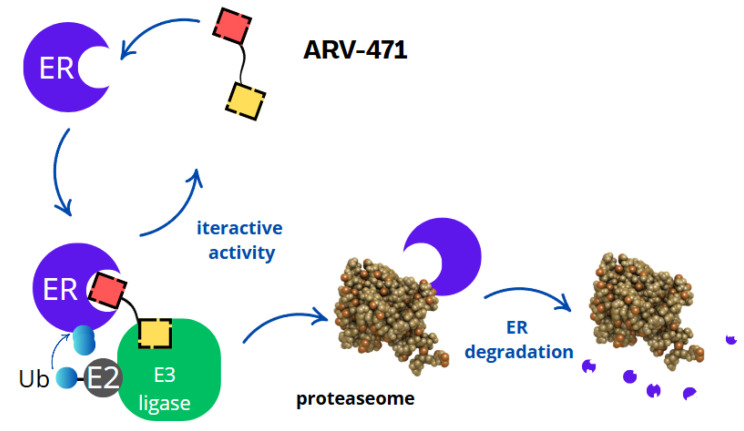
ARV-471 mechanism of action.

Figure 4 illustrates ARV-471’s distinct mechanism of action. ARV-471, a novel oral PROTAC drug which targets the oestrogen receptor, shows high efficacy in treating ER+ breast cancer in preclinical models. As monotherapy, ARV-471 significantly inhibits tumour growth. The PROTAC mechanism is based on the recruitment of ubiquitinating enzymes (e.g., E3 ligases), which attach ubiquitin to the target protein, marking it for degradation by proteasomes. In the case of ARV-471, the target is the oestrogen receptor, and the recruitment of the E3 ligase leads to its degradation. The oestrogen receptor (ER) is a nuclear protein that, upon binding with oestrogens, regulates the transcription of genes responsible for cell proliferation [39]. In ER+ breast cancer cells, the overexpression of this receptor is key for the growth and survival of cancer cells. Traditional therapies, such as aromatase inhibitors and selective oestrogen receptor modulators (SERMs), work by inhibiting ER activity or blocking its binding to oestrogens. However, these approaches have limitations, such as treatment resistance or partial inhibition of ER activity [40]. ARV-471 works in an entirely different way. Instead of blocking the oestrogen receptor, ARV-471 causes its degradation, thus eliminating its function in cancer cells. This mechanism can be broken down into several stages.

1. Binding to the oestrogen receptor—ARV-471 contains a ligand that specifically binds to the oestrogen receptor, marking it for degradation.
2. Recruitment of E3 ligase—ARV-471 acts as a bridge, connecting the oestrogen receptor with the E3 ligase, an enzyme responsible for tagging proteins with ubiquitin.
3. Ubiquitination and proteasomal degradation—the E3 ligase attaches ubiquitin to the oestrogen receptor, signalling its degradation by proteasomes, which break it down into amino acids.
4. Inhibition of cancer cell proliferation and induction of apoptosis—the degradation of the oestrogen receptor inhibits tumour growth by blocking gene transcription linked to cancer cell proliferation and inducing programmed cell death (apoptosis).
5. Synergistic action and therapeutic advantages—ARV-471 enhances the effects of CDK4/6 inhibitors (e.g., palbociclib) and PI3K/mTOR inhibitors (e.g., everolimus), while also offering efficacy in resistant cases, lower toxicity, and potential use in advanced breast cancer.

## 4. Clinical Trials

The first PROTAC was in the form of a peptide and was demonstrated in 2001. In 2008, the first set of small-molecule degraders was published. Between 2008 and 2018, numerous PROTACs unsuitable for oral administration were developed, demonstrating excellent biological activity both in vitro and in vivo [11,41]. Currently, the oral PROTAC degrader ARV-110 is in Phase II clinical trials, while ARV-471 has advanced to Phase III clinical trials. Table 3 shows how many clinical trials are ongoing and have been conducted using ARV-471. They are targeted against androgen receptors for the treatment of prostate cancer and oestrogen receptors for the treatment of breast cancer. The data comes from the clinical trial registry (clinicaltrials.gov) [42].

In preclinical studies, ARV-471 showed strong effectiveness in mice that had human ER+ breast cancer cells implanted. As a single treatment, ARV-471 greatly reduced tumour growth. Even greater efficacy was achieved when ARV-471 was used in combination with palbociclib or everolimus. The first clinical trial of ARV-471 began in 2019, marking a breakthrough in the development of PROTAC-based therapies. This study aimed to assess the safety, tolerability, and efficacy of ARV-471 in patients with advanced ER+ breast cancer. The results obtained in subsequent clinical trial phases confirmed the potential effectiveness of this drug, making it a promising therapeutic option for patients with ER+ breast cancer [43].

ARV-110 is an oral PROTAC targeting the androgen receptor (AR) and was designed as a potential therapy for patients with castration-resistant prostate cancer (CRPC). It is currently in Phase II clinical trials, which are evaluating its efficacy and safety in patients with advanced prostate cancer [44,45]. The clinical trials for ARV-110 began with Phase I, which aimed to assess the safety and tolerability of the drug in patients who had previously received standard anti-androgen therapies. The results of this phase indicated that ARV-110 has an acceptable safety profile and is well-tolerated. The drug then moved on to Phase II, where its effectiveness in reducing AR levels and inhibiting tumour progression is being investigated. The mechanism of action of ARV-110 is based on selective degradation of the androgen receptor [45,46]. The drug consists of two parts—one that binds to AR and another that recruits the E3 ligase responsible for attaching ubiquitin. This process marks AR for destruction by proteasomes, which could be a useful treatment for patients whose usual AR inhibitors do not work because of mutations or too much of the receptor. Preclinical results were promising—ARV-110 effectively degraded AR in several cell lines of prostate cancer and significantly reduced tumour size in mouse models [47]. Preliminary results from clinical trials also indicate the drug’s activity in patients with AR mutations, suggesting its potential as a new therapeutic option. Ongoing clinical trials are working to better understand how well ARV-110 works, both on its own and when used with other anti-androgen treatments. Positive results could pave the way for further clinical stages and potential approval of the drug for the treatment of advanced prostate cancer [42,48].

## 5. New Treatment Possibilities Using PROTACs

**Figure 5:**
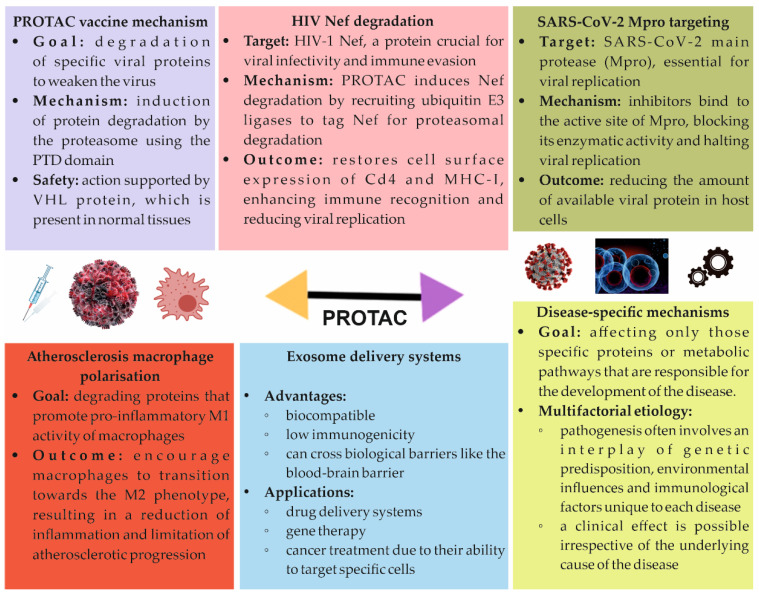
New treatment possibilities using PROTACs: PROTAC vaccine, HIV Nef degradation, SARS-CoV-2 Mpro targeting, atherosclerosis macrophage polarisation, exosome delivery systems, disease-specific mechanisms.

Recently, the dynamic development of PROTACs has enabled the creation of new molecules targeting a wide range of pathogenic proteins. An increasing number of studies indicate their effectiveness and safety, making them a promising alternative to conventional therapies. This chapter presents the latest research directions on PROTAC and their potential applications in the treatment of various diseases, with particular emphasis on oncology, neurology, and autoimmune disorders. In Figure 5, a diagram illustrating key new treatment opportunities using PROTACs is presented. Moreover, in Table 4, a presentation of the PROTACs used in the treatment of various types of cancer and the targets they act on is provided as potential treatments [49].

### 5.1. PROTAC Vaccine

In a conceptual study conducted by Si et al., researchers developed an innovative chimeric vaccine technology based on PROTACs, which utilises the host’s ubiquitin–proteasome system for the controlled degradation of influenza virus proteins [68]. The PROTAC vaccine works by adding a special part that can be broken down to the influenza virus M1 protein, which helps weaken the virus and triggers strong immune responses in animals like mice and ferrets. The researchers emphasise that this approach can be extended to the production of live attenuated vaccines against various pathogens, making it a promising tool in the fight against multiple infectious diseases [69].

In the context of existing influenza vaccines, such as inactivated vaccines, attenuated vaccines for low-temperature use, and recombinant vaccines, PROTAC vaccines exhibit unique advantages. Currently available influenza vaccines, although safe, often provide suboptimal protection. Live attenuated vaccines can be highly effective, but producing them through adaptive mutations from serial passaging is time-consuming and does not always yield safe viral strains. An example of this is the FluMist vaccine, which acquires debilitating mutations during prolonged culture, yet offers only limited efficacy due to the constant need to maintain six segments of the viral genome, complicating antigenic matching. In contrast, the PROTAC vaccine utilises a novel mechanism for directing viral proteins to the host’s ubiquitin–proteasome system, leading to their degradation and a weakening of viral replication. A main part of this method is the peptide ALAPYIP, which attaches to the E3 ubiquitin ligase von Hippel–Lindau (VHL) and triggers the breakdown of viral proteins in regular cells. For production purposes, this peptide also includes a cleavage site for the tobacco etch virus (TEV) protease (TEVp), which can be selectively separated by TEVp in modified cells. In studies, the PROTAC vaccine effectively replicated in MDCK.2 cells (Madin–Darby canine kidney cells) expressing TEVp, suggesting the possibility of large-scale production. In conventional MDCK.2 cells, the replication ability was drastically reduced, demonstrating effective attenuation. Tests on mice and ferrets showed that the virus was weakened and that there was a strong immune response, including antibodies and T cells. The PROTAC virus elicited higher antibody titers and T cell responses compared to conventional vaccines. The PROTAC technology offers an innovative approach to developing safe and effective vaccines that can overcome the limitations of conventional methods. These vaccines utilise degraded viral peptides generated during the proteasomal degradation process to elicit a strong immune response. Moreover, the reduced potential for immune escape makes PROTACs a promising technology for the future in combating rapidly evolving viruses [70,71].

### 5.2. PROTACs and HIV

The Nef genes in primate lentiviruses like HIV-1, HIV-2, and SIV produce special proteins that are important for the virus to replicate, survive, and cause AIDS. The HIV-1 Nef protein is made in large amounts soon after infection and works with different proteins in the host cells to make the virus more infectious and help it avoid the immune system [72]. Some of the best-characterised Nef partner proteins include the endocytic adaptor proteins AP-1 and AP-2, which are essential for downregulating the expression of major histocompatibility complex I (MHC-I), CD4, and other viral receptors, as well as the restriction factor SERINC5 Nef which also binds and activates non-receptor protein-tyrosine kinases and actin cytoskeleton regulators to promote transcription and release of the virus. Degradation of Nef may reduce the pathogenesis of HIV-1 and help in the reduction or elimination of persistent viral reservoirs. HIV-1 gene expression, including Nef, is maintained in individuals living with HIV, even under suppressive ART. Continuous production and release of Nef and other HIV-encoded proteins from latent reservoir cells may contribute to HIV-associated comorbidities, including cardiovascular and central nervous system disorders. There is a significant correlation between the ability of patient-derived Nef alleles to reduce MHC-I expression in vitro and the size of the reservoir in vivo, with larger viral reservoirs associated with increased MHC-I expression downregulation activity. Studies have demonstrated that Nef PROTAC effectively restores CD4 and MHC-I expression on the surface of T cells, suggesting that targeted degradation of Nef is likely to reverse the endocytosis of all cell surface receptors and restricting factors mediated by Nef. Duette et al. reported that Nef expression is essential for the survival of intact HIV-1 proviruses in effector memory T cells, which dominate as reservoir cells. The expression of the immune checkpoint receptor PD-1 on cell surfaces is also related to the survival of viral reservoirs in vitro and in vivo. Checkpoint-blocking antibodies, such as Keytruda (pembrolizumab), can induce latency reversal in individuals living with HIV and in non-human primates infected with SIV. Nef is important for HIV to make PD-1 appear on cell surfaces, which helps keep the hidden HIV-1 in the body by avoiding the immune system. Using PROTAC to specifically break down Nef could stop all of its functions, which might help wake up dormant HIV, boost the immune response, slow down the virus’s growth, and ultimately lower the number of hidden HIV cells [73].

### 5.3. COVID-19 Treatment

SARS-CoV-2, the virus responsible for the COVID-19 pandemic, employs various proteins for replication and spread within host cells. One of the key viral proteins is the main protease (Mpro), which plays a crucial role in the viral life cycle by processing long polypeptide chains into active viral proteins. Mpro inhibitors have been studied as potential therapies against COVID-19, but limitations related to their efficacy and the development of resistance have emerged. Research presented in the publication “Discovery of FIRST in-class PROTAC degraders of SARS-CoV-2 main protease” highlights the utilisation of PROTAC for the specific degradation of the viral protease Mpro. This novel therapeutic strategy aims not just to block the protein’s activity but to completely eliminate it from the cell, which may be more effective in preventing infection. PROTAC degraders designed to defend against Mpro act by binding to the viral protein, recruiting the ubiquitin complex. This process results in the ubiquitination of Mpro and its subsequent degradation by proteasomes, thereby reducing the amount of available viral protein in host cells. By this mechanism, PROTACs may more effectively limit viral replication compared to traditional inhibitors. In vitro studies have shown that degraded Mpro significantly reduces the virus’s ability to replicate in cells, potentially leading to faster symptom resolution and limited transmission. The application of PROTACs in COVID-19 treatment opens new therapeutic possibilities. As clinical research progresses, they may become an alternative to current treatment methods, particularly in the context of the risk of mutations and viral resistance to standard therapies. Consequently, PROTACs could also provide a foundation for future drugs against other viruses. PROTACs represent a ground-breaking approach in the treatment of viral infections, including COVID-19. The selective degradation of SARS-CoV-2 main protease using PROTACs is a new therapeutic strategy showing promising results in preclinical studies. With ongoing research and development of PROTAC technology, there is potential for the introduction of new, effective drugs to combat the pandemic and other viral diseases in the future [74].

### 5.4. Atherosclerosis Treatment

One of the most common causes of cardiovascular diseases, such as myocardial infarction, is thrombosis and the rupture of atherosclerotic plaques. Statins are the most frequently prescribed medications, yet these drugs have numerous side effects, including hepatotoxicity, nephrotoxicity, and various neurological and neurocognitive disorders [75,76,77]. Therefore, there is a critical need for new solutions characterised by fewer adverse effects. Macrophages, which are key components of the immune response, play a dual role in atherosclerosis—possibly supporting or inhibiting the development of atherosclerotic lesions. M1 macrophages are pro-inflammatory and contribute to the inflammatory response, while M2 macrophages have anti-inflammatory effects and support tissue repair. Thus, the drive to switch macrophage phenotype from M1 to M2 may represent a promising therapeutic strategy in treating atherosclerosis [78].

In the context of atherosclerosis treatment, PROTAC technology is gaining importance as a tool for selective protein degradation. PROTACs consist of ligands that bind to the target protein, ligands that recruit E3 ubiquitin ligases, and a linker. This allows not only the inhibition of protein activity but its complete degradation. This strategy can be applied to modulate macrophage phenotype, influencing inflammation-related functions and immune responses. Research on the application of PROTAC in atherosclerosis treatment has shown that specific degraders can target key proteins associated with the inflammatory process in macrophages. By degrading proteins that promote pro-inflammatory M1 activity, it is possible to encourage macrophages to transition towards the M2 phenotype, resulting in a reduction in inflammation and limitation in atherosclerotic progression. In particular, studies have demonstrated that PROTACs can affect signalling pathways, such as the NF-κB pathway, which is responsible for regulating inflammatory responses. By degrading specific regulatory proteins, PROTACs can effectively alter macrophage profiles and reduce inflammation in blood vessels. An additional advantage of using PROTACs is the potential for their combination with nanotechnology. Nanoparticles can be used for the targeted delivery of PROTACs to sites of atherosclerotic lesions, increasing their bioavailability and minimising side effects. By employing nanotechnology, it becomes possible to precisely target macrophages within atherosclerotic plaques, significantly enhancing the efficacy of the therapy. The introduction of PROTAC into the treatment of atherosclerosis could open new therapeutic avenues [79].

### 5.5. The Use of PROTAC in the Treatment of RNA Viral Infections

PROTAC technology is gaining increasing recognition as a promising therapeutic strategy in the fight against RNA viral infections, such as hepatitis B and C, Zika virus, Ebola virus, and diseases caused by coronaviruses. PROTACs are innovative molecules that combine elements of ligands binding to a target protein with those targeting E3 ubiquitin ligases, enabling the selective degradation of chosen proteins. A key aspect of the PROTAC function is their ability to degrade both viral proteins and host proteins essential for viral replication, leading to the inhibition of pathogen replication and a reduction in the viral load within the patient’s body. However, the success of PROTAC therapies often hinges on the effective delivery of these complex molecules to the appropriate target cells. In recent years, there has been growing attention towards exosomes as potential carriers for PROTACs. Exosomes are microscopic vesicles released by various cells that play a crucial role in intercellular communication. They contain a variety of biomolecules, including proteins, lipids, and RNA, and their natural ability to easily penetrate cell membranes makes them ideal candidates for use as drug delivery platforms. The mechanism of PROTAC delivery via exosomes involves their modification for the loading of appropriate PROTAC molecules. In this process, exosomes are first isolated from cell cultures and then incubated with PROTACs. As a result of interactions between the proteins present on the surface of the exosomes and the PROTAC molecules, the latter are absorbed and loaded. These modified exosomes can then be utilised in therapy, reaching target cells where the PROTACs can induce the degradation of viral proteins as well as host proteins that facilitate viral replication [80,81].

Exosomes as carriers offer several advantages. First, their biocompatibility and natural ability to transport biomolecules reduce the risk of degradation by the host immune system, thus enhancing their potential efficacy. Second, due to their small size and liposomal structure, exosomes can easily penetrate cell membranes, delivering PROTACs directly into virus-infected cells. Research on the use of exosomes for PROTAC delivery in the context of RNA viral infections is still in its early stages, but preliminary results are promising. The application of this strategy may not only enhance the therapeutic efficacy of PROTACs but also minimise their side effects by directing drugs straight to infectious cells. With further research and the development of delivery technologies using exosomes, PROTACs may become a new important tool in combating viral infections and treating diseases with high epidemic risk. In the future, we can expect that innovative approaches based on exosomes will significantly impact the effectiveness of viral therapies, contributing to improved patient quality of life and paving new pathways in drug development [80].

### 5.6. Treatment of Alzheimer’s Disease

In recent years, PROTAC technologies have been actively investigated as potential tools for the therapy of Alzheimer’s disease. One of the key targets in this therapeutic strategy is glycogen synthase kinase 3 beta (GSK-3β), which plays a significant role in the pathogenesis of Alzheimer’s disease, including the phosphorylation of tau protein, leading to the formation of pathological neurofibrillary tangles [82]. Research has shown that using PROTACs to degrade GSK-3β could be an effective strategy to reduce the levels of this kinase, thereby inhibiting the processes related to neuronal degeneration [83].

Tau proteins are essential microtubule-associated proteins found predominantly in neurones within the central nervous system. Their primary function is to stabilise microtubules, which are crucial for maintaining cellular structure and facilitating intracellular transport. These proteins exist in six highly soluble isoforms, produced through alternative splicing of the MAPT gene. In the context of neurodegenerative diseases such as Alzheimer’s, tau proteins undergo hyperphosphorylation, resulting in the formation of insoluble aggregates known as neurofibrillary tangles. These tangles interfere with neuronal function and communication. Diseases characterised by abnormal tau accumulation, including Alzheimer’s disease, Pick’s disease, and progressive supranuclear palsy, are collectively referred to as tauopathies. Tau-targeting PROTACs are designed to selectively degrade tau proteins by recruiting an E3 ubiquitin ligase to the tau protein, facilitating its ubiquitination and subsequent degradation by the proteasome. Unlike traditional inhibitors, PROTACs act catalytically, meaning a single PROTAC molecule can degrade multiple tau proteins. This approach offers a promising therapeutic strategy for treating tauopathies by reducing tau protein levels and mitigating neurofibrillary tangle formation. Several tau-targeting PROTACs are currently being investigated in preclinical and clinical trials to evaluate their safety, efficacy, and therapeutic potential. One of the significant challenges in developing PROTACs for tau proteins is ensuring they can effectively reach and degrade tau proteins within the brain, given the presence of the blood–brain barrier. Achieving high selectivity for tau proteins while minimising off-target effects is also crucial for the success of tau-targeting PROTAC. Recent studies have indicated that PROTAC can selectively degrade hyperphosphorylated tau, demonstrating potential for therapeutic applications in Alzheimer’s disease. Companies like Arvinas are actively working on developing PROTACs targeting tau and other proteins related to neurodegenerative diseases. Tau-targeting PROTACs represent a cutting-edge approach in the fight against neurodegenerative diseases, offering hope for more effective treatments [84].

Further investigations into the use of PROTACs for protein interactions in the context of transthyretin (TTR) as a novel strategy in developing drugs against Alzheimer’s disease indicate that modifying PROTACs to target TTR enables the selective degradation of the proteins that contribute to neurodegeneration. TTR is a transport protein that is crucial in the pathology of Alzheimer’s disease, as its dysfunction can lead to amyloid accumulation and disturbances in the amyloid β metabolism [85]. Research suggests that PROTACs may facilitate the degradation of these pathological proteins and modulate protein interactions, thereby opening new avenues for therapeutic design [86].

In another study, the action of PROTACs was examined concerning the degradation of the epidermal growth factor receptor (EGFR), which is associated with cancer cell proliferation and neurodegeneration. Alterations in EGFR function are linked to both cancer and Alzheimer’s disease, making the degradation of this protein using PROTAC a promising therapeutic strategy. Consequently, employing PROTACs to degrade EGFR may contribute to reversing the pathological pathways involved in these diseases [87].

Research findings indicate that the application of PROTACs in the therapy of Alzheimer’s disease may lead to a reduction in clinically significant proteins associated with neuronal degeneration, such as GSK-3β, and improve synaptic function. Furthermore, improvements in cognitive function have been observed in animal models treated with PROTACs, suggesting that this approach may significantly influence the development of therapeutic strategies against Alzheimer’s disease. It is proposed that further research into these technologies could contribute to breakthroughs in the treatment of neurodegenerative diseases [83,85,87].

### 5.7. PROTAC Technology to Combat Stress Hormone Receptor Activation

The application of PROTAC technology in the context of combating the activation of stress hormone receptors, such as the glucocorticoid receptor (GR), presents an innovative approach for the therapy of stress-related disorders and hormonal responses [88]. PROTACs represent a novel strategy that leverages the mechanisms of protein ubiquitination, enabling the selective degradation of target proteins via the proteasome. In the case of stress hormone receptors, various isoforms of GR that are involved in the pathogenesis of disorders such as depression, anxiety, and adaptive disorders can serve as therapeutic targets. Research has demonstrated that using PROTACs to degrade GR can effectively reduce its transcriptional activity, leading to decreased expression of glucocorticoid-dependent genes that are associated with the adverse effects of stress. In experimental contexts, synthesised PROTACs have shown the ability to selectively degrade GR in human cells, and the application of these compounds in animal models has contributed to improvements in the behavioural parameters related to stress response [89].

It is suggested that active degradation of GR may not only lower the body’s response to stress but also modulate metabolism, neuroplasticity, and immune responses. Such approaches could lead to innovative pharmacological therapies that would enable the treatment of stress-related disorders in a manner that is more controlled and targeted than traditional methods that act by blocking receptor activity. Further research in this area may provide new, effective therapeutic strategies for treating conditions associated with hormonal dysregulation and stress response [88].

### 5.8. Atopic Dermatitis

The treatment of atopic dermatitis (AD) is a complex process where new therapeutic approaches, such as PROTAC-based degraders, are emerging as a promising strategy. The mechanism of action of degrading Janus kinase (JAK1 and JAK2) proteins focuses on the precise regulation of signalling pathways that are critical in the pathogenesis of AD, particularly in the context of aberrant immune responses. PROTAC-based degraders operate by selectively degrading target proteins through ubiquitination and proteolysis mechanisms. In the case of JAK1 and JAK2, which play central roles in cytokine signalling transduction, the degradation of these kinases enables the modulation of their excessive activity, which leads to the inflammatory states observed in AD. PROTACs designed to target JAK1 and JAK2 combine a ligand specific for these kinases with an E3 ligase, allowing their effective degradation by the proteasome. When a PROTAC is introduced into the cell, the ligand binds to JAK1 or JAK2, inducing a conformational change that exposes interaction sites for the E3 ligase. This results in the post-translational modification of the protein by the attachment of ubiquitin residues, initiating a degradation cascade that leads to the removal of the protein by the proteasome. The reduction in JAK1 and JAK2 levels in cells disrupts cytokine signalling, translating to decreased inflammation and an alleviation of symptoms associated with AD, such as itching, dryness, and skin inflammation. Furthermore, the degradation of JAK1 and JAK2 may also influence other signalling pathways related to immunology and inflammation, such as IL-4, IL-13, and IL-31 pathways, which are recognised for their roles in the pathogenesis of AD. By reducing the activity of these proteins, it may be possible to simultaneously affect the expression of pro-inflammatory genes and regulate immune cells, which is crucial for controlling inflammatory responses. The use of JAK1/JAK2 degraders based on PROTAC as new therapeutic strategies in AD may also reduce the risk of the undesirable side effects associated with traditional JAK inhibitors, which often act in a non-specific manner and can lead to immunosuppression or other adverse reactions. In the context of AD, the degradation of specific target proteins using PROTACs opens a new pathway for effective, targeted, and safe therapeutic intervention. However, such approaches require further clinical studies and mechanistic investigations to fully understand their potential and application in AD therapy [90].

### 5.9. Non-Alcoholic Fatty Liver Disease

PROTAC technology represents a novel approach in the treatment of non-alcoholic fatty liver disease (NAFLD) and other metabolic disorders, focusing on the targeted degradation of proteins that play critical roles in the pathogenesis of these conditions [91,92]. PROTACs are bifunctional molecules that link target proteins to E3 ligases, resulting in their degradation in the proteasome. A key mechanism of action for PROTACs is their ability to induce ubiquitination of the target protein through reversible binding to the appropriate E3 ligase proteins, leading to a significant decrease in protein concentration within the cells. In the context of fatty liver disease, the degradation of proteins that regulate lipid metabolism, such as SREBP (sterol regulatory element-binding proteins) and other factors associated with inflammatory responses, is particularly promising, as these proteins are crucial for the progression of NAFLD. By reducing the levels of SREBP, PROTACs can alter the lipid profile of hepatocytes, decreasing lipid accumulation in the liver and improving various markers of liver function. Therefore, controlling the expression of genes involved in lipid metabolism using PROTACs appears to be a promising avenue for research in NAFLD therapy. Additionally, the degradation of pro-inflammatory proteins using PROTACs may help to reduce the inflammatory response, which is a key factor in the progression of NAFLD to more advanced conditions such as hepatitis or cirrhosis [93,94]. Strengthening the connection between SREBP degradation by PROTACs and potential clinical outcomes or improvements in NAFLD is crucial. Degradation of SREBP by PROTACs can lead to reduced lipogenesis in the liver, which is essential for treating NAFLD. SREBPs are key transcription factors regulating genes involved in cholesterol, fatty acid, and triglyceride biosynthesis. Their overactivity is linked to NAFLD pathogenesis, causing excessive fat accumulation, inflammation, and hepatocyte damage. By decreasing SREBP levels, PROTACs can reduce fat accumulation in the liver, lower inflammation, and improve liver function. Preclinical studies have shown that inhibiting SREBP can improve clinical outcomes, such as reducing liver fibrosis and the risk of hepatocellular carcinoma [95].

It is noteworthy that the incorporation of PROTACs into therapies for fatty liver disease is not only aimed at removing pathogenic proteins but also allows for selective targeting of specific molecular pathways, which is essential for minimising side effects. Through such a precise strategy, PROTACs may offer new therapeutic possibilities to combat fatty liver disease and the accompanying inflammation. Given the promising results in preclinical models, further research into the application of PROTACs in the context of NAFLD is necessary to understand their full therapeutic potential and the likelihood of implementation in clinical practice. The PROTAC technology, with its ability to facilitate targeted protein degradation, appears to be a promising strategy in the fight against liver diseases, including fatty liver disease, which may lead to the development of new, effective therapeutic methods in the future [96].

### 5.10. Huntington’s Disease

In the context of Huntington’s disease, research on the application of PROTACs is focused on the primarily problematic mutant huntingtin protein (mHtt) [97]. In publications concerning mHtt, PROTACs are presented as an innovative therapeutic approach aimed at the selective degradation of the mutated form of huntingtin, potentially significantly impacting disease progression [86,98]. mHtt, resulting from mutations in the HTT gene, leads to the accumulation of pathological protein forms that are responsible for neurodegeneration and a range of clinical proteasomal complexes, enabling its degradation. This mechanism may mitigate the negative effects of mHtt while preserving the functionality of healthy forms of huntingtin. In 2017, Tomoshige et al. developed two PROTACs (PROTAC 19 and PROTAC 20) that targeted the mHtt. These molecules were created by combining mHtt inhibitors (benzothiazole derivatives named BTA and PDB) with a ligand of the E3 ligase VHL using polyethylene glycol-like compounds of various lengths. Studies showed that PROTAC 19 and PROTAC 20 effectively reduced mHtt levels in the ubiquitin–proteasome system in primary cells derived from patients with Huntington’s disease, as well as in HeLa cells transfected with exon-1 mHtt with long polyQ repeats. Furthermore, PROTAC 19 demonstrated the ability to reduce mHtt aggregates in these cells. The strategy of targeting protein aggregates appears to be a promising approach in developing PROTAC directed against misfolded proteins [96,99].

### 5.11. Treatment of Immune Disorders

IRAK4, associated with the interleukin-1 receptor, plays a crucial role in innate immune processes. It belongs to the IRAK family, which includes four serine-threonine kinases: IRAK4, IRAK1, IRAK2, and IRAK-M. Research has shown that the loss of function or deficiency of IRAK4 increases susceptibility to pathogens, while excessive activation is linked to certain autoimmune diseases. Although some inhibitors have been tested in clinical trials to block kinases, they have not achieved the desired effects, as reports indicate that non-kinase functions or scaffolding capabilities play a more significant role in certain cell types [100].

To address these challenges, researchers from GlaxoSmithKline developed a PROTAC targeting IRAK4, based on PF-06650833, to eliminate all protein functions, including kinase-dependent and scaffolding roles, which could lead to broader pharmacological effects than traditional inhibitors. In their study, they tested the E3 ligase ligands VHL, CRBN, and IAP, finding that only the VHL-based PROTAC was effective in degrading IRAK4. A fully carbon-based hydrophobic chain proved to be a better linker than hydrophilic PEG due to its permeability. Following modifications to include a more rigid, polar spirocyclic pyrimidine, compound 70 was produced, demonstrating improved solubility, higher DC50 values (151 nM in peripheral blood mononuclear cells and 36 nM in skin fibroblasts), and lower in vitro clearance in liver microsomes. However, the PROTAC (compound 70) did not show a distinct pharmacological profile compared to the inhibitor (PF-06650833) in this study, indicating that further investigation is needed to fully understand IRAK4 biology [101].

## 6. New Solutions

The conjugation of PROTAC with an antibody, known as a “degrader-antibody conjugate” (DAC), represents a cutting-edge approach to improving targeted protein degradation in various disease contexts. By merging the precise targeting of antibodies with the strong protein breakdown ability of PROTACs, this method provides a hopeful way to make treatments more effective, especially for cancer and other hard-to-treat diseases. This innovative approach not only increases the selectivity of PROTACs but also helps to improve their pharmacokinetic properties, reducing off-target effects and enhancing their therapeutic potential [102]. The DAC technology is an example of a new approach for developing PROTAC structures. Table 5 presents emerging PROTAC technologies achieved by modifying the main elements of the structure but keeping the multifunctional principle of building these compounds.

### 6.1. Antibody–PROTAC

The antibody–PROTAC approach is an innovative technology that combines the PROTAC protein degradation mechanism with precise antibody delivery. This strategy can significantly improve tissue and cell specificity by minimising side effects and increasing the therapeutic window of action [103]. Research indicates that antibody–PROTAC conjugates, like trastuzumab-PROTAC, can specifically break down proteins in HER2+ cells, showing that they target certain tissues. Experiments, like those carried out by the Tate group, show that trastuzumab-PROTAC links a BRD4 degrader with trastuzumab, causing BRD4 protein to break down specifically in HER2+ cells [104].

### 6.2. Aptamer-PROTAC Conjugates

The aptamer-PROTAC is another novel technology that combines aptamers, single-stranded nucleic acids with complex structures, with PROTACs [105,106]. Aptamers offer exceptional specificity and affinity for target proteins while improving membrane permeability and water solubility. Studies have shown that aptamer-PROTAC can effectively degrade BET proteins, such as BRD4, in MCF-7 breast cancer cells, while reducing toxicity and increasing targeting specificity [104,107].

### 6.3. Dual-Target PROTAC

Another innovation is dual-target PROTAC, designed to degrade two different proteins simultaneously. This strategy can counteract cancer-cell drug resistance, which often results from the compensatory activation of alternative signalling pathways. An example is the compound DP-V-4, which degrades both EGFR and PARP, offering a potential cancer treatment that may be more effective than therapies targeting a single protein [106,108].

### 6.4. Folate-Caged PROTAC

The folate-caged PROTAC technology introduces folic acid groups into PROTAC molecules, allowing their specific release in cancer cells that express the folate receptor (FOLR1). This strategy minimises toxicity in normal tissues. An example is Folate-ARV-771, which degrades the BRD4 protein in cancer cells with high folate expression while showing minimal activity in normal cells [109,110].

### 6.5. TF–PROTAC

Transcription factors (TFs) are challenging to target due to the absence of traditional binding pockets for small molecules. However, TF–PROTACs offer a novel strategy for degrading these proteins, potentially leading to new cancer therapies. Small-molecule inhibitors are often ineffective against TFs, but TF–PROTAC technology can overcome these limitations, providing new therapeutic opportunities [111,112].

In summary, modern PROTAC technologies, including antibody–PROTAC, aptamer-PROTAC, dual-target PROTAC, folate-caged PROTAC, and TF–PROTAC, represent significant advancements in precise and effective cancer treatment. Each of these technologies offers unique benefits that can enhance therapeutic efficacy and minimise side effects, opening new avenues in oncology [42].

In 2018, a ribonuclease-targeting chimera (RIBOTAC) was introduced. RIBOTAC can specifically bring in the natural ribonuclease RNase L to a chosen RNA target, which helps to effectively remove RNA through the cell’s process of breaking down nucleic acids. Recently, Disney’s team refined their research by converting Dovitinib, a tyrosine kinase receptor (RTK) inhibitor, into a small RIBOTAC molecule. This modified molecule demonstrated 2500 times greater selectivity and reduced toxicity in degrading pathogenic RNA. Animal studies have shown that this molecule has significant anti-cancer effects [113].

### 6.6. PhosphoTAC

Phosphorylation is one of the key post-translational protein modifications. Disruptions in the phosphorylation of critical cellular proteins are strongly associated with many diseases, including cancer and Alzheimer’s disease. The phosphorylation process is dynamic and reversible, regulated by both kinases and phosphatases. Kinases catalyse protein phosphorylation, while phosphatases are responsible for dephosphorylation. Initially, research focused on developing small inhibitors for protein kinases and phosphatases, but this strategy has limitations. First, designing selective kinase inhibitors is challenging. Second, because inhibitors often target the ATP-binding sites of kinases, they may also interact with ATP-binding sites of other kinases, leading to potential off-target toxicity. Third, mutations in kinase-binding sites can lead to drug resistance. Therefore, there is a need for further improvement in strategies to treat phosphorylation-related disorders [37].

**Figure 6:**
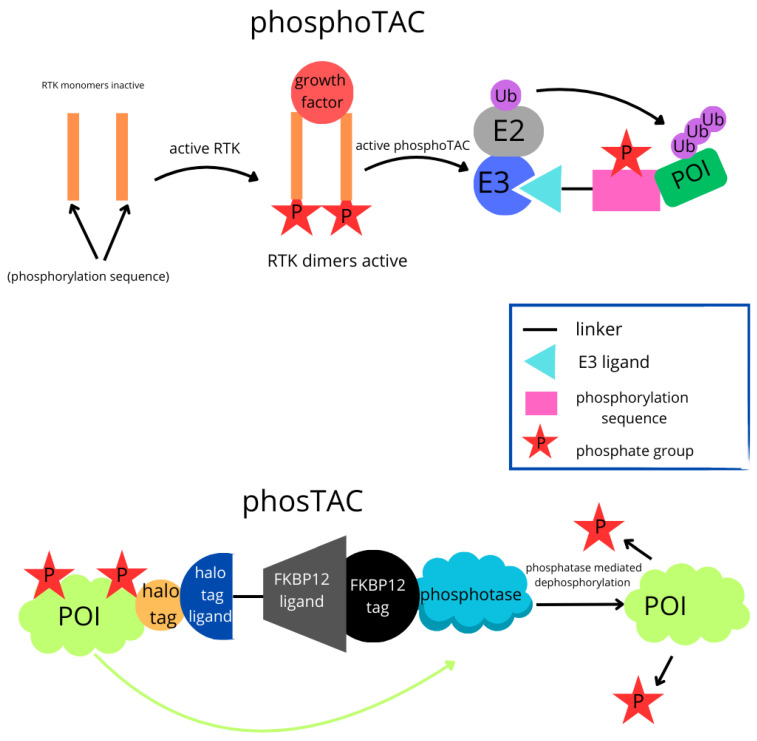
PhosphoTAC and PhosTAC mechanisms of action for and key differences between these technologies.

Based on the core principles of PROTACs, Crews’ research group designed chimeric molecules targeting phosphorylation-dependent proteolysis (phosphoPROTAC). The distinctive feature of phosphoPROTAC is that its activity depends on the activation of receptor tyrosine kinases (RTK) in the signalling pathway. Only phosphoPROTAC activated by RTK can degrade the target protein. Specific RTKs, such as TrkA and ERBB2/ERBB3, form dimers and autophosphorylate tyrosine residues upon activation. Phosphorylated tyrosine sequences of TRKA and ERBB3 proteins are conjugated to Von Hippel–Lindau (VHL) E3 ubiquitin ligase ligands via an aminohexanoic acid linker, forming phosphoPROTAC. After phosphorylation by activated RTK, two phosphoPROTACs become active and recruit the receptor substrate factor 2α (FRS2α) and phosphatidylinositol-3 kinase (PI3K), leading to their degradation via ubiquitination. Both phosphoPROTACs demonstrate the ability to inhibit their respective RTK signalling pathways both in vitro and in vivo. Additionally, a mutation of tyrosine to phenylalanine deactivates phosphoTAC, showing that their activation is entirely dependent on kinase phosphorylation. Furthermore, the stimulation of unrelated growth factor receptors does not induce target protein degradation, confirming the specificity of phosphoPROTACs within signalling pathways. In summary, PhosphoTAC and PhosTAC are innovative technologies used for targeted protein modulation, each with distinct mechanisms. PhosphoTAC technology targets phosphorylated proteins for degradation. PhosphoTAC bind to phosphorylated proteins, which are often involved in signalling pathways and disease processes. Upon binding, they recruit E3 ubiquitin ligases, leading to the ubiquitination and subsequent degradation of the phosphorylated protein by the proteasome. In contrast, PhosTAC aims to dephosphorylate proteins rather than degrade them. This mechanism involves a ligand that binds to the POI fused with a HaloTag, and another ligand that recruits a phosphatase fused with an FKBP12 (F36V) tag. The phosphatase then catalyses the dephosphorylation of the POI [114]. Key differences are that PhosphoTAC focuses on degrading phosphorylated proteins by recruiting E3 ubiquitin ligases, contrary to PhosTAC which targets proteins for dephosphorylation by recruiting phosphatases. Figure 6 illustrates the mechanisms of action for PhosphoTAC and PhosTAC and the key differences between these technologies.

### 6.7. PhosTAC

In 2021, the concept of phosphorylation-targeting chimeras (PhosTAC) was introduced. This is a more precise method of manipulating the activity of target proteins through dephosphorylation rather than degradation. The mechanism of action of PhosTAC is similar to that of PROTACs, with the difference being that PhosTAC recruits a phosphatase instead of an E3 ligase. As a result, PhosTAC does not degrade the target protein but dephosphorylates its substrates, thus regulating the function of the protein. This offers a more precise way of controlling the activity of the target protein. In the study, a phosphatase was conjugated with FKBP12 (F36V), and the substrate protein with Halo Tag. At the same time, the FKBP12 (F36V) ligand was combined with the Halo Tag ligand (chloroalkane) via poly(ethylene glycol) (PEG) linkers, forming a PhosTAC complex. It was demonstrated that this PhosTAC complex effectively mediates the dephosphorylation of substrates such as PDCD4 and FOXO3a. Unlike traditional PROTAC-induced protein degradation, PhosTAC allows for more precise manipulation of specific protein functions, offering new possibilities for protein regulation [115,116,117,118].

### 6.8. Photocaged PROTAC

PROTACs hold promising clinical prospects due to their ability to target protein degradation. However, there is a risk of on-target and off-target effects that may lead to toxic side effects when PROTACs act on healthy tissues. To address this, many studies focus on developing spatially and temporally controlled PROTACs. One of the first solutions was photocaged PROTACs, such as opto-PROTACs and pc-PROTACs. Photocages are molecular groups that can be photodegraded, allowing for PROTAC-activity control through light. In this mechanism, the photocage acts as a cage, deactivating the PROTACs [80,119,120]. Upon light exposure, the cage is photodegraded, releasing the active PROTACs. An example is opto-PROTACs, developed in 2019 by the team of Jian Jin and Wenyi Wei. In their research, they added a photolabile group to pomalidomide, blocking its binding to the CRBN E3 ligase. Under UVA light, the photolabile group was degraded, releasing the active pomalidomide. Similarly, dBET1 and ALK inhibitors were modified to become photocontrolled PROTACs, named Opto-DBET1 and Opto-DALK. These modifications resulted in lower toxic side effects and more controlled catalytic activity. Overall, opto-PROTACs may be more suitable for precision medicine than traditional PROTACs. Additionally, based on the known BRD2-4 degrader dBET1, Xue and colleagues developed photoactivated PROTACs known as pc-PROTACs. They introduced BBP into dBET1, which reduced its binding to BRD4. While the compound was inactive in the dark, after 3 min of exposure to 365 nm light, it effectively degraded BRD4 in Ramos cells. The compound was also used in zebrafish, where it induced BRD4 degradation during embryogenesis. Although photocaged PROTACs can induce protein degradation, they have one drawback: the process is irreversible, and once activated, their inactivation cannot be controlled. To overcome this limitation, photoswitchable PROTACs such as PHOTACs, AZO-PROTAC, and photoPRO isomerisation were developed, allowing reversible activation or deactivation under light of different wavelengths. For example, Pfaff and colleagues reported photoswitchable PROTACs (photoPROTAC) containing ortho-F4-azobenzene linkers. Reynders and colleagues developed PHOTACs targeting BET family proteins (BRD2, 3, 4) and FKBP12. Jin and colleagues designed Azo-PROTACs for degrading BCR-ABL fusion and ABL proteins in K562 myeloid leukaemia cells. These studies enabled reversible protein degradation, making them more versatile tools in molecular biology [121,122].

### 6.9. CLIPTAC

PROTAC molecules typically consist of two covalently linked ligands, which often have a high molecular weight and relatively poor solubility, drug-like properties, and bioavailability. “Click chemistry,” introduced by Sharpless’ lab, has been used to describe a series of chemical reactions with mild reaction conditions, high product efficiency, and high selectivity. Today, it serves as a theoretical foundation for covalent modifications and self-assembly under physiological conditions. In 2016, Heightman TD proposed the concept of CLIPTAC, in which the PROTAC was split into two precursor drugs: a POI–half-linker and a half-linker–ligand of the E3 ubiquitin ligase. These two precursors were administered separately and subsequently self-assembled intracellularly, forming a complete PROTAC structure and inducing degradation of the POI. While this method faces challenges, particularly with the efficiency of intracellular reactions, it remains an intriguing attempt to improve the bioavailability of PROTACs. By splitting the molecule into smaller, more drug-like components that can self-assemble, the aim is to enhance the delivery and efficacy of these therapeutic agents within cells [33,123,124].

### 6.10. PROTAC Applications in the Prevention of Virus Threats or Pandemics

Viruses have long posed one of the greatest threats to public health worldwide [71,125]. Throughout human history, numerous viruses have caused epidemics and pandemics, resulting in a significant death toll and affecting the lives of millions [126]. One of the most notable examples is the Spanish flu virus (H1N1), which led to the deaths of 50 to 100 million people globally between 1918 and 1919 [127]. In contemporary times, one of the most dangerous viruses is HIV, responsible for the AIDS epidemic. Since its discovery in the 1980s, AIDS has claimed the lives of over 32 million individuals [128]. In recent years, the COVID-19 pandemic, caused by the SARS-CoV-2 virus, has emerged as a global health crisis. Since its onset at the end of 2019 and into 2020, by the end of 2023, COVID-19 has resulted in the deaths of over 6.9 million people worldwide. The scale of the pandemic and its impact on health systems, economies, and societies underscored the importance of preparedness and rapid response to emerging viral threats. The search for new vaccines and antiviral therapies is not only crucial for combating current threats but also for preventing future pandemics. As Dr. Anthony Fauci, director of the National Institute of Allergy and Infectious Diseases, stated, “We must develop more innovative strategies to combat viruses before they become a global threat”. Examples illustrate that the rapid development and global deployment of vaccines, as seen in the case of COVID-19, can significantly reduce infection and death rates, accelerating a return to normality. However, this process is not without challenges. Financial resources, international coordination, and scientific innovations are imperative. The effectiveness of vaccines and antiviral therapies may be hindered by viral mutations, as seen with variants of SARS-CoV-2, highlighting the need for continuous monitoring and quick adaptation of virus combat strategies. The development of new technologies, including mRNA-based vaccines, opens new possibilities for the prevention and treatment of viral diseases. Moderna and Pfizer-BioNTech, two companies that developed mRNA vaccines against COVID-19, have become pioneers in this field, and their successes indicate the vast potential of these technologies for combating other viruses. “The future of pandemic response may depend on our ability to rapidly react to new viral threats and our commitment to research that will help us better understand and control these pathogens,” emphasised Dr. Richard Hatchett, CEO of the Coalition for Epidemic Preparedness Innovations (CEPI), in one of his statements, in summary, viruses remain a significant threat to global health, and history shows that pandemics can have dramatic consequences. Protecting humanity requires ongoing efforts to seek new vaccines and antiviral therapies. Investment in science, support for innovation, and global collaboration are essential for effectively combating current and future viral threats [129].

## 7. Study Limitations and Challenges

PROTACs function catalytically, meaning a single molecule can degrade multiple target proteins. This characteristic allows the use of lower doses compared to traditional inhibitors. The sustained degradation effect means that PROTACs can be administered less frequently, potentially improving patient adherence. Some PROTACs that target essential genes may have a narrow therapeutic index, necessitating careful dose optimisation to balance efficacy and toxicity. Regular monitoring of biomarkers can help adjust dosing to ensure optimal therapeutic outcomes.

Combining PROTACs with other therapies, such as chemotherapy or immunotherapy, can enhance efficacy and potentially reduce required doses. Implementing sequential dosing strategies can help manage resistance and improve overall treatment outcomes. Continued research into advanced drug delivery systems will be crucial to overcoming pharmacokinetic challenges and improving the clinical efficacy of PROTACs. Refining the design of PROTACs to enhance their stability, solubility, and specificity will be key to their success in clinical settings. While current research is heavily focused on cancer, PROTACs have potential applications in other diseases, such as neurodegenerative disorders and viral infections [130].

Despite the promising therapeutic potential of PROTAC technology, its structural and physicochemical characteristics pose significant challenges to drug development. One of the fundamental obstacles stems from the inherent architecture of PROTAC molecules, which typically include at least two ligands connected by a linker. This results in high molecular weight and complex chemical structures, which often violate Lipinski’s rule of five guidelines that help predict oral bioavailability. High molecular weight and polarity make it hard for the drug to pass through cell membranes and be absorbed, which limits how well it spreads in the body and reduces its effectiveness, especially when taken by mouth, which is the most common way to give drugs [130]. To provide a clearer perspective on these limitations, Table 6 presents a comparison of PROTACs with other targeted protein-degradation strategies, including molecular glues and hydrophobic tagging [131,132,133].

Moreover, PROTAC compounds are characterised by large topological polar surface areas due to multiple polar functional groups, which further hinder their ability to cross biological membranes and physiological barriers. These properties can negatively impact solubility, permeability, and metabolic stability, making it difficult to achieve sufficient oral bioavailability. To address these shortcomings, researchers have explored various strategies, including modifying linker characteristics (length, rigidity, and hydrophobicity), reducing ligand size, and implementing prodrug approaches. For instance, the addition of lipophilic moieties has been shown to enhance solubility and oral uptake [130,134]. Another key issue is poor permeability. In vitro models have repeatedly shown limited cell penetration by PROTAC molecules, often compounded by low solubility and high nonspecific binding, which leads to inadequate recovery and unreliable permeability data. To improve cellular uptake, structural optimisation of the linker—particularly the use of shorter or more hydrophobic linkers—can improve lipid membrane penetration. Nevertheless, these modifications must be carefully balanced to avoid compromising the molecule’s degradation efficiency or target affinity. Additionally, the “hook effect”, a bell-shaped concentration–response relationship, presents a unique pharmacodynamic challenge. At higher concentrations, excessive binding to either the target protein or E3 ligase can prevent the formation of the necessary ternary complex, ultimately reducing degradation activity. This phenomenon complicates dose optimisation and highlights the need for precise pharmacokinetic and pharmacodynamic modelling [135].

Another important consideration is the potential for the development of drug resistance. As with other targeted therapies, prolonged exposure to PROTACs may induce cellular adaptation or mutations in the target protein or E3 ligase, reducing degradation efficiency over time. Moreover, the complexity of PROTAC molecules and their intracellular mechanisms may lead to the activation of compensatory pathways or evasion strategies by cancer cells and other disease-related systems. Off-target effects and systemic toxicity also remain major safety concerns. Due to their high potency and irreversible degradation mechanism, unintended interactions with non-target proteins could lead to unpredictable biological outcomes or adverse effects. There is currently a lack of standardised validation protocols for assessing on-target vs. off-target activity, selectivity, and proteasome dependence. Finally, the synthetic complexity of PROTACs significantly increases production costs and development timelines. Each step of synthesis must be optimised, and the purification of intermediates can be labour-intensive. Additionally, the pharmaceutical formulation of PROTAC, including the development of a stable solid form suitable for clinical use, can pose further difficulties. In summary, while PROTAC technology holds considerable promise, numerous limitations, ranging from poor pharmacokinetics and bioavailability to toxicity, resistance development, and manufacturing hurdles, must be carefully addressed through continued innovation in drug design, delivery systems, and validation standards [45].

## 8. Materials and Methods

The literature analysis was conducted in accordance with the PRISMA guidelines [136].

Data sources and search strategy:

A literature search was performed using the Science Direct, Scopus, and PubMed databases to compile a preselected set of publications essential to the topic. A predefined set of keywords was utilised, with “PROTAC” as the primary term. Boolean operator AND was employed to incorporate secondary terms for search refinement, including “bioavailability”, “pharmacokinetics”, “in vitro assessment”, “in vivo studies”, “therapeutic efficacy”, and “pharmaceutical innovations”.

The inclusion criteria for this study comprised peer-reviewed articles available in full text, published between 2021 and 2025 in reputable journals, studies involving human or animal models, articles in English, and research presenting either quantitative or qualitative data pertinent to the topic. Exclusion criteria removed studies that were not relevant to PROTAC applications, non-peer-reviewed sources, publications outside the specified timeframe, and articles with inadequate methodology or ambiguous results. Records duplicated across various databases were consolidated into a single record.

This search strategy aimed to ensure a thorough and accurate selection of literature that reflects recent advancements in the field. Ultimately, 136 publications were selected.

## 9. Summary

The publication regarding PROTACs focuses on the innovative applications of this technology in the treatment of various diseases, including cancer. PROTACs represent an advanced therapeutic strategy that enables the selective degradation of proteins, thereby opening new possibilities in pharmacotherapy. Moreover, PROTACs show significant potential in treating different types of tumours by targeting oncogenic proteins that are critical for the growth and survival of cancer cells. This approach not only allows for the elimination of harmful proteins but also induces an immune response, which can further enhance treatment efficacy. Worth noting, PROTACs can also be applied in the context of vaccines, as they facilitate the removal of viral proteins, thereby supporting the immune response. In addition, regarding atherosclerosis, PROTACs could be utilised to regulate proteins associated with lipid metabolism and inflammation. Concerning RNA viral infections, the PROTAC technology can be directed at viral proteins, thus aiding the development of new antiviral therapies. On the other hand, in the context of Huntington’s Disease, PROTACs may be employed to degrade pathogenic proteins, potentially alleviating symptoms and slowing disease progression. Furthermore, PROTACs could also play a role in treating non-alcoholic fatty liver disease by regulating the proteins responsible for lipid metabolism. In the case of Huntington’s disease, PROTACs can be designed to degrade harmful multi-protein aggregates. These innovative formulations aim to enhance the effectiveness of applications in more complex biological systems and increase the stability and bioavailability of drugs. In summary, PROTACs present innovative possibilities in the treatment of many diseases, and the development of new technologies and formulations could significantly influence the future of therapy in oncology and other fields of medicine.

The development of PROTACs involves high costs, mainly due to the complex processes of synthesis and pharmacokinetic testing. Unlike traditional small molecule inhibitors, which have more established and predictable development pathways, PROTACs require additional research into their unique properties and mechanisms of action. These costs may affect the availability of these therapies for patients, as higher development costs may lead to higher drug prices on the market.

## Figures and Tables

**Table 1:** Principles of linker design in PROTACs.

Principle	Description
Linker Length	PEG increases solubility and compatibility with biological systems.
Linker Composition	Hydrophobic linkers can improve membrane permeability, which is crucial for targeting intracellular proteins. Alkyl chains can improve membrane permeability. Hydrophobic linkers may have solubility issues.
Attachment Points	Attachment points of the linker to the ligand are crucial for proper formation of the ternary complex Optimisation of length and attachment points can significantly impact protein degradation efficiency.
Linker Permeability	Chemical composition and flexibility of the linker are key for forming folded conformations, which correlate with high cellular permeability.

**Table 2:** Comparison of PROTAC with traditional inhibitors.

Dimension	PROTAC	Traditional Inhibitors
Mechanism	Targeted protein degradation via the ubiquitin–proteasome system	Inhibition of protein function by binding to active sites
Catalytic Efficiency	Catalytic; one PROTAC molecule can degrade multiple target proteins	Non-catalytic; requires continuous binding to inhibit protein function
Target Scope	Can target “undruggable” proteins lacking active sites	Limited to proteins with well-defined active sites
Clinical Advantages	Lower doses are required, the degradation is sustained, and the off-target effects are reduced	Effective for proteins with accessible active sites and well-established methods
Resistance Mechanisms	Can overcome resistance due to mutations in active sites	Resistance often arises from mutations in the protein’s active site
Dosing	Lower doses are required, as PROTACs act catalytically; one PROTAC molecule can degrade multiple targets	Requires continuous presence at high concentrations to maintain therapeutic effect
Dosing Frequency	The sustained degradation effect has resulted in a reduced dosing frequency	Frequent dosing is necessary to ensure the inhibitor remains bound to the target protein

**Table 3:** PROTAC ARV-471 in clinical trials.

Clinical Trial Number	Phase of Clinical Trial	Study Status	Conditions	Enrolment
NCT05930925	Phase 1	Completed	Healthy	12
NCT05732428	Phase 1	Completed	Breast cancer	9
NCT04072952	Phase 1 Phase 2	Active, not recruiting	Breast cancer	217
NCT05501769	Phase 1	Active, not recruiting	Breast cancer	32
NCT05548127	Phase 1 Phase 2	Active, not recruiting	Breast cancer	37
NCT05652660	Phase 1	Completed	Healthy	12
NCT05573555	Phase 1 Phase 2	Recruiting	Breast cancer	47
NCT05673889	Phase 1	Completed	Healthy	24
NCT06125522	Phase 1 Phase 2	Recruiting	Breast cancer	67
NCT05463952	Phase 1	Active, not recruiting	Breast neoplasms	6
NCT05549505	Phase 2	Completed	Breast cancer	152
NCT05909397	Phase 3	Active, not recruiting	Breast cancer	1180
NCT05654623	Phase 3	Active, not recruiting	Advanced breast cancer	624
NCT06347861	Phase 1	Completed	Healthy	52
NCT06645938	Phase 1	Recruiting	Healthy	12
NCT05538312	Phase 1	Completed	Healthy	12
NCT06206837	Phase 1 Phase 2	Recruiting	Breast cancer	65
NCT06256510	Phase 1	Completed	Healthy participants	15
NCT06005688	Phase 1	Completed	Healthy participants	12
NCT01042379	Phase 2	Recruiting	Angiosarcoma Breast cancer Breast neoplasms Breast tumours	5000
NCT06275841	Phase 1	Completed	Healthy	12

**Table 4:** Presentation of PROTACs used in the treatment of various types of cancer and the targets they act on.

Indications	Degrader	Target	Ref.
Breast cancer	ARV-471	ER	[50]
	AC-682	ER	[51]
Prostate cancer	ARV-110	AR	[48]
	CC-94676	AR	[52]
	HP-518	AR	[53]
	AC0176	AR	[54]
Cancer and solid tumours	CFT-1946	BRAF-V600	[55]
	RNK-05047	BRD4	[56]
Multiple myeloma	CFT-7455	IKZF1/3	[57]
Non-small cell lung cancer	CFT8919	EGFR	[58]
B-cell lymphoma	HSK29116	BTK	[59]
B-cell malignant tumour	NX-5948	BTK	[60]
	NX-2127	BTK	[61]
Synovial sarcoma	FHD-609	BRD9	[62]
Advanced synovial sarcoma	CFT8634	BRD9	[63]
Pancreatic cancer and solid tumours	ASP3082	KRAS G12D	[64]
B cell malignancy	BGB-16673	BTK	[65]
Haematologic cancers	AC0676	BTK	[66]
Soft tissue sarcoma	CFT8634	BRD9	[63]
Solid and blood tumour	DT2216	BCL-XL	[67]

**Table 5:** Emerging PROTAC technologies.

Technology Type	Schematic Representation	Therapeutic Implications
Antibody-PROTAC	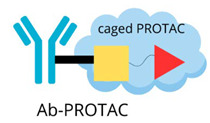	The precise targeting ability of antibodies with the protein-degradation function of PROTAC: - selective breakdown of disease-causing proteins in specific tissues or cell types, - improving therapeutic accuracy, - minimising unintended side effects.
Aptamer-PROTAC Conjugates	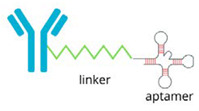	The precise binding ability of aptamers and the protein-degrading function of PROTAC to selectively degrade disease-associated proteins: - improves therapeutic accuracy, - minimises unintended effects, - holds promise for treating a range of diseases, such as cancer.
Dual-Target PROTAC	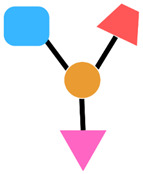	This dual engagement strategy is designed to simultaneously engage two different proteins: - enhancing the degradation of disease-related targets, - increasing therapeutic efficacy, - reducing the likelihood of resistance, - offering potential for treating multifactorial diseases like cancer.
Folate-Caged PROTAC	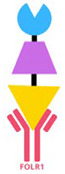	This targeted approach utilises folate receptors, which are highly expressed in many cancers, to selectively deliver and degrade disease-causing proteins: - enhancing therapeutic precision, - minimising off-target effects, - holding promise for improving cancer treatment outcomes.
TF-PROTAC	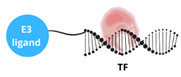	This approach enables the degradation of transcription factors that lack small molecule binding sites and target transcription factors by combining a DNA oligonucleotide with an E3 ligase ligand: - enhancing therapeutic precision, - reducing off-target effects, - offering potential for treating cancers and other diseases.
PhosphoTAC	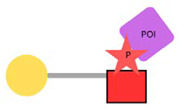	This approach is designed to degrade specific proteins by leveraging phosphorylation. When activated by receptor tyrosine kinases (RTKs), PhosphoTAC recruits proteins such as FRS2α and PI3K, leading to their ubiquitination and subsequent degradation.
PhosTAC	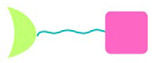	Small bifunctional molecules that promote dephosphorylation by bringing a phosphatase close to the target protein.
Photocaged PROTAC	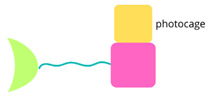	This approach is designed to degrade target proteins upon exposure to specific wavelengths of light: - precise spatial and temporal control of protein degradation, making it a promising strategy for treating diseases like cancer, - minimising off-target effects, - reducing toxicity.
CLIPTAC	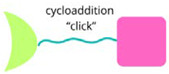	Utilise click chemistry to assemble bifunctional molecules within cells, enhancing targeted protein degradation.

**Table 6:** Comparison of PROTACs with molecular glues and hydrophobic tagging.

Aspect	PROTAC	Molecular Glues	Hydrophobic Tagging
Mechanism	Link target protein to E3 ubiquitin ligase for ubiquitination and degradation	Stabilize interaction between E3 ubiquitin ligase and target protein for degradation	Attach hydrophobic tag to target protein, causing misfolding and degradation
Advantages	High specificity Catalytic nature, requiring lower doses	Simplicity Broad applicability	Simplicity Versatility
Specificity	High specificity	Moderate specificity	Lower specificity
Complexity	High complexity in synthesis and optimisation	Moderate complexity	Lower complexity
Mechanism Type	Relies on ubiquitin–proteasome system	Relies on ubiquitin–proteasome system	Does not rely on ubiquitin–proteasome system

## References

[1] Bray F., Laversanne M., Sung H., Ferlay J., Siegel R.L., Soerjomataram I., Jemal A. (2024). Global cancer statistics 2022: GLOBOCAN estimates of incidence and mortality worldwide for 36 cancers in 185 countries. CA A Cancer J. Clin..

[2] Siegel R.L., Giaquinto A.N., Jemal A. (2024). Cancer statistics. CA A Cancer J. Clin..

[3] Siegel R.L., Miller K.D., Jemal A. (2019). Cancer statistics. CA A Cancer J. Clin..

[4] Holohan C., Van Schaeybroeck S., Longley D.B., Johnston P.G. (2013). Cancer drug resistance: An evolving paradigm. Nat. Rev. Cancer.

[5] Burslem G.M., Crews C.M. (2020). Proteolysis-Targeting Chimeras as Therapeutics and Tools for Biological Discovery. Cell.

[6] Pettersson M., Crews C.M. (2019). PROteolysis TArgeting Chimeras (PROTACs)—Past, present and future. Drug Discov. Today Technol..

[7] He X., Weng Z., Zou Y. (2024). Progress in the controllability technology of PROTAC. Eur. J. Med. Chem..

[8] Li X., Song Y. (2020). Proteolysis-targeting chimera (PROTAC) for targeted protein degradation and cancer therapy. J. Hematol. Oncol..

[9] Zou Y., Ma D., Wang Y. (2019). The PROTAC technology in drug development. Cell Biochem. Funct..

[10] Neklesa T.K., Winkler J.D., Crews C.M. (2017). Targeted protein degradation by PROTACs. Pharmacol. Ther..

[11] Bondeson D.P., Crews C.M. (2017). Targeted Protein Degradation by Small Molecules. Annu. Rev. Pharmacol. Toxicol..

[12] Li D., Yu D., Li Y., Yang R. (2022). A bibliometric analysis of PROTAC from 2001 to 2021. Eur. J. Med. Chem..

[13] Yan S., Zhang G., Luo W., Xu M., Peng R., Du Z., Liu Y., Bai Z., Xiao X., Qin S. (2024). PROTAC technology: From drug development to probe technology for target deconvolution. Eur. J. Med. Chem..

[14] Zhang S., Lai Y., Pan J., Saeed M., Li S., Zhou H., Jiang X., Gao J., Zhu Y., Yu H. (2024). PROTAC Prodrug-Integrated Nanosensitizer for Potentiating Radiation Therapy of Cancer. Adv. Mater..

[15] Kuriyama M., Wang C.F., Nagase T., Sohma Y., Kanai M., Hori Y., Tomita T. (2025). Proteolytic therapeutic modalities for amyloidoses: Insights into immunotherapy, PROTAC, and photo-oxygenation. Neurotherapeutics.

[16] Wang Z., Zhang D., Inuzuka H., Wei W. (2025). PROTAC technology for prostate cancer treatment. Acta Mater. Medica.

[17] Monsen P.J., Bommi P.V., Grigorescu A.A., Lauing K.L., Mao Y., Berardi P., Zhai L., Ojo O., Penco-Campillo M., Koch T. (2025). Rational Design and Optimization of a Potent IDO1 Proteolysis Targeting Chimera (PROTAC). J. Med. Chem..

[18] Simpson L.M., Glennie L., Brewer A., Zhao J.-F., Crooks J., Shpiro N., Sapkota G.P. (2022). Target protein localization and its impact on PROTAC-mediated degradation. Cell Chem. Biol..

[19] Takano R., Ohoka N., Kurohara T., Arakawa N., Ohgane K., Inoue T., Yokoo H., Demizu Y. (2025). Clozapine as an E3 Ligand for PROTAC Technology. ACS Med. Chem. Lett..

[20] Sheng X.-Y., Wu S.-H., Li B.-L., Li X.-N., Wu H.-S., Cao J. (2021). Advances in the optimization of the linker in proteolysis-targeting chimeras (PROTAC). Acta Pharm. Sinica.

[21] Ma N., Yang T., Mukhaleva E., Wei W., Vaidehi N. (2025). BPS2025-Innovative strategies in PROTAC design: Evaluating linker efficiency through protein frustration analysis. Biophys. J..

[22] Troup R.I., Fallan C., Baud M.G.J. (2020). Current strategies for the design of PROTAC linkers: A critical review. Explor. Target. Anti-tumor Ther..

[23] Poongavanam V., Peintner S., Abeje Y., Kölling F., Meibom D., Erdelyi M., Kihlberg J. (2025). Linker-Determined Folding and Hydrophobic Interactions Explain a Major Difference in PROTAC Cell Permeability. ACS Med. Chem. Lett..

[24] Abeje Y.E., Wieske L.H.E., Poongavanam V., Maassen S., Atilaw Y., Cromm P., Lehmann L., Erdelyi M., Meibom D., Kihlberg J. (2024). Impact of Linker Composition on VHL PROTAC Cell Permeability. J. Med. Chem..

[25] Kumar H., Sobhia M.E. (2024). Interplay of PROTAC Complex Dynamics for Undruggable Targets: Insights into Ternary Complex Behavior and Linker Design. ACS Med. Chem. Lett..

[26] Li L., Mi D., Pei H., Duan Q., Wang X., Zhou W., Jin J., Li D., Liu M., Chen Y. (2020). In vivo target protein degradation induced by PROTACs based on E3 ligase DCAF15. Signal Transduct. Target. Ther..

[27] Ward C.C., Kleinman J.I., Brittain S.M., Lee P.S., Chung C.Y.S., Kim K., Petri Y., Thomas J.R., Tallarico J.A., McKenna J.M. (2019). Covalent Ligand Screening Uncovers a RNF4 E3 Ligase Recruiter for Targeted Protein Degradation Applications. ACS Chem. Biol..

[28] Belcher B.P., Ward C.C., Nomura D.K. (2021). Ligandability of E3 Ligases for Targeted Protein Degradation Applications. Biochemistry.

[29] Medvar B., Raghuram V., Pisitkun T., Sarkar A., Knepper M.A. (2016). Comprehensive database of human E3 ubiquitin ligases: Application to aquaporin-2 regulation. Physiol. Genom..

[30] Danishuddin, Jamal M.S., Song K.-S., Lee K.-W., Kim J.-J., Park Y.-M. (2023). Revolutionizing drug targeting strategies: Integrating artificial intelligence and structure-based methods in PROTAC development. Pharmaceuticals.

[31] Palomba T., Baroni M., Cross S., Cruciani G., Siragusa L. (2023). ELIOT: A platform to navigate the E3 pocketome and aid the design of new PROTACs. Chem. Biol. Drug Des..

[32] Zagidullin A., Milyukov V., Rizvanov A., Bulatov E. (2020). Novel approaches for the rational design of PROTAC linkers. Explor. Target. Anti-Tumor Ther..

[33] Wang S., He F., Tian C., Sun A. (2024). From PROTAC to TPD: Advances and Opportunities in Targeted Protein Degradation. Pharmaceuticals.

[34] Weerakoon D., Carbajo R.J., De Maria L., Tyrchan C., Zhao H. (2022). Impact of PROTAC Linker Plasticity on the Solution Conformations and Dissociation of the Ternary Complex. J. Chem. Inf. Model..

[35] Zhong L., Li Y., Xiong L., Wang W., Wu M., Yuan T., Yang W., Tian C., Miao Z., Wang T. (2021). Small molecules in targeted cancer therapy: Advances, challenges, and future perspectives. Signal Transduct. Target. Ther..

[36] Backes A., Zech B., Felber B., Klebl B., Müller G. (2008). Small-molecule inhibitors binding to protein kinases. Part I: Exceptions from the traditional pharmacophore approach of type I inhibition. Expert Opin. Drug Discov..

[37] Xiao M., Zhao J., Wang Q., Liu J., Ma L. (2022). Recent Advances of Degradation Technologies Based on PROTAC Mechanism. Biomolecules.

[38] Graham H. (2022). The mechanism of action and clinical value of PROTACs: A graphical review. Cell. Signal..

[39] Schott A.F., Hurvitz S., Ma C., Hamilton E., Nanda R., Zahrah G., Hunter N., Tan A.R., Telli M., Mesias J.A. (2023). Abstract GS3-03: GS3-03 ARV-471, a PROTAC® estrogen receptor (ER) degrader in advanced ER-positive/human epidermal growth factor receptor 2 (HER2)-negative breast cancer: Phase 2 expansion (VERITAC) of a phase 1/2 study. Cancer Res..

[40] Snyder L.B., Flanagan J.J., Qian Y., Gough S.M., Andreoli M., Bookbinder M., Cadelina G., Bradley J., Rousseau E., Chandler J. (2021). Abstract 44: The discovery of ARV-471, an orally bioavailable estrogen receptor degrading PROTAC for the treatment of patients with breast cancer. Cancer Res..

[41] Li Y., Yang J., Aguilar A., McEachern D., Przybranowski S., Liu L., Yang C.-Y., Wang M., Han X., Wang S. (2018). Discovery of MD-224 as a First-in-Class, Highly Potent, and Efficacious Proteolysis Targeting Chimera Murine Double Minute 2 Degrader Capable of Achieving Complete and Durable Tumor Regression. J. Med. Chem..

[42] Han X., Sun Y. (2022). Strategies for the discovery of oral PROTAC degraders aimed at cancer therapy. Cell Rep. Phys. Sci..

[43] Gough S.M., Flanagan J.J., Teh J., Andreoli M., Rousseau E., Pannone M., Bookbinder M., Willard R., Davenport K., Bortolon E. (2024). Oral Estrogen Receptor PROTAC Vepdegestrant (ARV-471) Is Highly Efficacious as Monotherapy and in Combination with CDK4/6 or PI3K/mTOR Pathway Inhibitors in Preclinical ER+ Breast Cancer Models. Clin. Cancer Res..

[44] Nguyen T.-T., Kim J.W., Choi H.-I., Maeng H.-J., Koo T.-S. (2022). Development of an LC-MS/MS Method for ARV-110, a PROTAC Molecule, and Applications to Pharmacokinetic Studies. Molecules.

[45] Benowitz A.B., Scott-Stevens P.T., Harling J.D. (2022). Challenges and Opportunities for In Vivo PROTAC Delivery. Futur. Med. Chem..

[46] Ma L., Han X. (2025). Bavdegalutamide (ARV-110): A potent PROTAC androgen receptor degrader for the treatment of metastatic-castration resistant prostate cancer. Drug Discovery Stories.

[47] Vetma V., O’Connor S., Ciulli A. (2024). Development of PROTAC Degrader Drugs for Cancer. Annu. Rev. Cancer Biol..

[48] Neklesa T., Snyder L.B., Willard R.R., Vitale N., Pizzano J.A., Gordon D., Bookbinder M., Macaluso J., Dong H., Ferraro C. (2019). ARV-110: An oral androgen receptor PROTAC degrader for prostate cancer. J. Clin. Oncol..

[49] Grigoreva T.A., Novikova D.S., Melino G., Barlev N.A., Tribulovich V.G. (2024). Ubiquitin recruiting chimera: More than just a PROTAC. Biol. Direct.

[50] Flanagan J.J., Qian Y., Gough S.M., Andreoli M., Bookbinder M., Cadelina G., Bradley J., Rousseau E., Willard R., Pizzano J. (2019). Abstract P5-04-18: ARV-471, an oral estrogen receptor PROTAC degrader for breast cancer. Cancer Res..

[51] Lloyd M.R., Wander S.A., Hamilton E., Razavi P., Bardia A. (2022). Next-generation selective estrogen receptor degraders and other novel endocrine therapies for management of metastatic hormone receptor-positive breast cancer: Current and emerging role. Ther. Adv. Med Oncol..

[52] Rathkopf D., Patel M., Choudhury A., Rasco D., Lakhani N., Hawley J., Srinivas S., Aparicio A., Narayan V., Runcie K. (2025). Safety and clinical activity of BMS-986365 (CC-94676), a dual androgen receptor ligand-directed degrader and antagonist, in heavily pretreated patients with metastatic castration-resistant prostate cancer. Ann. Oncol..

[53] Azad A., Gurney H., Underhill C., Horvath L., Voskoboynik M., Li X., King I., Shao L., Dai Y., Perabo F. (2024). Preliminary data from a dose-escalation phase 1 study with HP518, an AR PROTAC degrader: Safety, tolerability, pharmacokinetics (PK), and first assessment of anti-tumor activity in patients (Pts) with metastatic castration-resistant prostate cancer (mCRPC). Am. Soc. Clin. Oncol..

[54] Li X., Mu P. (2024). Restoring our ubiquitination machinery to overcome resistance in cancer therapy. Oncoscience.

[55] Scott J.S., Michaelides I.N., Schade M. (2024). Property-based optimisation of PROTACs. RSC Med. Chem..

[56] Peng X., Hu Z., Zeng L., Zhang M., Xu C., Lu B., Tao C., Chen W., Hou W., Cheng K. (2024). Overview of epigenetic degraders based on PROTAC, molecular glue, and hydrophobic tagging technologies. Acta Pharm. Sin. B.

[57] Kong N.R., Jones L.H. (2023). Clinical Translation of Targeted Protein Degraders. Clin. Pharmacol. Ther..

[58] Park E.S., Ahn J.Y., Baddour J., Chaturvedi P., Chiu M.I., Cole K.S., Crystal A.S., Duplessis M., Fisher S.L., Good A.C. (2021). Preclinical Evaluation of CFT8919 as a Mutant Selective Degrader of EGFR with L858R Activating Mutations for the Treatment of Non-Small Cell Lung Cancer; Keystone Esymposium Targeting Protein Degradation: From Small Molecules to Complex Organelles.

[59] Li J., Xu W., Yan P., Cao Y., Hu M., Daley W. (2023). Abstract CT128: Phase 1 study of HSK29116, a Bruton tyrosine kinase (BTK) proteolysis-targeting chimera (PROTAC) agent, in patients with relapsed or refractory B-cell malignancies. Cancer Res..

[60] Robbins D.W., Noviski M., Rountree R., Tan M., Brathaban N., Ingallinera T.E., Karr D., Kelly A., Konst Z., Ma J. (2021). Nx-5948, a Selective Degrader of BTK with Activity in Preclinical Models of Hematologic and Brain Malignancies. Blood.

[61] Mato A.R., Wierda W.G., Ai W.Z., Flinn I.W., Tees M., Patel M.R., Patel K., O’Brien S., Bond D.A., Roeker L.E. (2022). NX-2127-001, a first-in-human trial of NX-2127, a Bruton’s tyrosine kinase-targeted protein degrader, in patients with relapsed or refractory chronic lymphocytic leukemia and B-cell malignancies. Blood.

[62] Livingston J.A., Blay J.-Y., Trent J., Valverde C., Agulnik M., Gounder M., Le Cesne A., McKean M., Wagner M.J., Stacchiotti S. (2025). A Phase I Study of FHD-609, a Heterobifunctional degrader of Bromodomain-containing Protein 9, in patients with advanced synovial sarcoma or SMARCB1-deficient tumors. Clin. Cancer Res..

[63] Jackson K.L., Agafonov R.V., Carlson M.W., Chaturvedi P., Cocozziello D., Cole K., Deibler R., Eron S.J., Good A., Hart A.A. (2022). Abstract ND09: The discovery and characterization of CFT8634: A potent and selective degrader of BRD9 for the treatment of SMARCB1-perturbed cancers. Cancer Res..

[64] Tolcher A.W., Park W., Wang J.S., Spira A.I., Janne P.A., Lee H.-J., Gill S., LoRusso P., Herzberg B., Goldman J.W. (2023). Trial in progress: A phase 1, first-in-human, open-label, multicenter, dose-escalation and dose-expansion study of ASP3082 in patients with previously treated advanced solid tumors and KRAS G12D mutations. Am. Soc. Clin. Oncol..

[65] Wu Y., Meibohm B., Zhang T., Hou X., Wang H., Sun X., Jiang M., Zhang B., Zhang W., Liu Y. (2024). Translational modelling to predict human pharmacokinetics and pharmacodynamics of a Bruton’s tyrosine kinase-targeted protein degrader BGB-16673. Br. J. Pharmacol..

[66] Patel M., Tees M., Khan N., Awan F., Bond D., Xu Q., Brown G., Zhang H., Woyach J. (2024). AC676, an Orally Bioavailable BTK Chimeric Degrader in Patients With B-cell Malignancies. Clin. Lymphoma Myeloma Leuk..

[67] He Y., Koch R., Budamagunta V., Zhang P., Zhang X., Khan S., Thummuri D., Ortiz Y.T., Zhang X., Lv D. (2020). DT2216—A Bcl-xL-specific degrader is highly active against Bcl-xL-dependent T cell lymphomas. J. Hematol. Oncol..

[68] Si L., Shen Q., Li J., Chen L., Shen J., Xiao X., Bai H., Feng T., Ye A.Y., Li L. (2022). Generation of a live attenuated influenza A vaccine by proteolysis targeting. Nat. Biotechnol..

[69] Zhang C., Hou J., Li Z., Shen Q., Bai H., Chen L., Shen J., Wang P., Su Y., Li J. (2025). PROTAR Vaccine 2.0 generates influenza vaccines by degrading multiple viral proteins. Nat. Chem. Biol..

[70] Li Z., Bai H., Xi X., Tian W., Zhang J.Z., Zhou D., Si L. (2022). PROTAC vaccine: A new way to live attenuated vaccines. Clin. Transl. Med..

[71] Ahmad H., Zia B., Husain H., Husain A. (2023). Recent advances in PROTAC-based antiviral strategies. Vaccines.

[72] Luo D., Luo R., Wang W., Deng R., Wang S., Ma X., Pu C., Liu Y., Zhang H., Yu S. (2024). Discovery of L15 as a novel Vif PROTAC degrader with antiviral activity against HIV-1. Bioorganic Med. Chem. Lett..

[73] Emert-Sedlak L.A., Tice C.M., Shi H., Alvarado J.J., Shu S.T., Reitz A.B., Smithgall T.E. (2024). PROTAC-mediated degradation of HIV-1 Nef efficiently restores cell-surface CD4 and MHC-I expression and blocks HIV-1 replication. Cell Chem. Biol..

[74] Alugubelli Y.R., Xiao J., Khatua K., Kumar S., Sun L., Ma Y., Ma X.R., Vulupala V.R., Atla S., Blankenship L.R. (2024). Discovery of First-in-Class PROTAC Degraders of SARS-CoV-2 Main Protease. J. Med. Chem..

[75] Björkegren J.L., Lusis A.J. (2022). Atherosclerosis: Recent developments. Cell.

[76] Libby P., Buring J., Badimon L., Hansson G., Deanfield J., Bittencourt M., Tokgozoglu L., Lewis E. (2019). Atherosclerosis. Nat. Rev. Dis. Primers.

[77] Lee S.-E., Chang H.-J., Sung J.M., Park H.-B., Heo R., Rizvi A., Lin F.Y., Kumar A., Hadamitzky M., Kim Y.J. (2018). Effects of statins on coronary atherosclerotic plaques: The PARADIGM study. JACC Cardiovasc. Imaging.

[78] Huang J.-H., Huang C.-J., Yu L.-N., Guan X.-L., Liang S.-W., Li J.-H., Liang L., Wei M.-Y., Zhang L.-M. (2023). Bioinspired PROTAC-induced macrophage fate determination alleviates atherosclerosis. Acta Pharmacol. Sin..

[79] Ma Y., Yang X., Ning K., Guo H. (2024). M1/M2 macrophage-targeted nanotechnology and PROTAC for the treatment of atherosclerosis. Life Sci..

[80] Mukerjee N., Maitra S., Ghosh A., Alexiou A., Thorat N.D. (2024). Exosome-mediated PROTAC delivery for treatment of RNA viral infections and zoonosis. Drug Discov. Today.

[81] Mukerjee N., Mukherjee D. (2025). PROTAC-based therapeutics for targeting HPV oncoproteins in head and neck cancers. Nano TransMed.

[82] Wei J., Wang J., Zhang J., Yang J., Wang G., Wang Y. (2022). Development of inhibitors targeting glycogen synthase kinase-3β for human diseases: Strategies to improve selectivity. Eur. J. Med. Chem..

[83] Guardigni M., Pruccoli L., Santini A., De Simone A., Bersani M., Spyrakis F., Frabetti F., Uliassi E., Andrisano V., Pagliarani B. (2023). PROTAC-Induced Glycogen Synthase Kinase 3β Degradation as a Potential Therapeutic Strategy for Alzheimer’s Disease. ACS Chem. Neurosci..

[84] Pradeepkiran J.A., Reddy P.H. (2021). Phosphorylated tau targeted small-molecule PROTACs for the treatment of Alzheimer’s disease and tauopathies. Biochim. Et Biophys. Acta (BBA)-Mol. Basis Dis..

[85] Ciccone L., Tonali N., Nencetti S., Orlandini E. (2021). Application of PROTAC strategy to TTR-Aβ protein-protein interaction for the development of Alzheimer’s disease drugs. Neural Regen. Res..

[86] Kong D., Meng L., Lin P., Wu G. (2025). Advancements in PROTAC-based therapies for neurodegenerative diseases. Future Med. Chem..

[87] Kargbo R.B. (2019). Treatment of Cancer and Alzheimer’s Disease by PROTAC Degradation of EGFR. ACS Med. Chem. Lett..

[88] Gazorpak M., Hugentobler K.M., Paul D., Germain P.-L., Kretschmer M., Ivanova I., Frei S., Mathis K., Rudolf R., Barrenechea S.M. (2023). Harnessing PROTAC technology to combat stress hormone receptor activation. Nat. Commun..

[89] Geiger T.M., Walz M., Meyners C., Kuehn A., Dreizler J.K., Sugiarto W.O., Maciel E.V.S., Zheng M., Lermyte F., Hausch F. (2024). Entdeckung eines potenten PROTAC ermöglicht die gezielte Ausschaltung der Gerüstfunktionen von FKBP51. Angew. Chem..

[90] Wu J., Li L., Zhu Q., Zhang T., Miao F., Cui Z., Dong G., Tai Z., Chen Z. (2024). JAK1/JAK2 degraders based on PROTAC for topical treatment of atopic dermatitis. Biomed. Pharmacother..

[91] Colleoni A., Fassi E., Miluzio A., Albani M., Lecchi D., Tempra G., De Amici M., Grazioso G., Biffo S., Matera C. Targeted degraders of eIF6: A novel strategy to remodulate liver pathological lipidic metabolism. Proceedings of the Merck Young Chemists’ Symposium (MYCS).

[92] Colleoni A., Fassi E., Miluzio A., Tempra G., Albani M., De Amici M., Grazioso G., Biffo S., Matera C. Exploiting the Potential of Computational Approaches in Medicinal Chemistry: CADD of Novel eIF6 Binders for the Development of anti-HCC PROTACs. Proceedings of the WIDEnzymes.

[93] Qi M., Zhong H., Cheng Z., Chen S., Xiao H., Shang J., Chen L., Sun J. (2023). Discovery of NAFLD-Improving Agents by Promoting the Degradation of Keap1. J. Med. Chem..

[94] Wang Y., Zheng J., Long Y., Wu W., Zhu Y. (2024). Direct degradation and stabilization of proteins: New horizons in treatment of nonalcoholic steatohepatitis. Biochem. Pharmacol..

[95] Winzker M., Friese A., Koch U., Janning P., Ziegler S., Waldmann H. (2020). Development of a pdeδ-targeting PROTACs that impair lipid metabolism. Angew. Chem..

[96] Wang C., Zhang Y., Xing D., Zhang R. (2021). PROTACs technology for targeting non-oncoproteins: Advances and perspectives. Bioorganic Chem..

[97] Tashima T. (2023). Proteolysis-Targeting Chimera (PROTAC) Delivery into the Brain across the Blood-Brain Barrier. Antibodies.

[98] Farrell K., Jarome T.J. (2021). Is PROTAC technology really a game changer for central nervous system drug discovery?. Expert Opin. Drug Discov..

[99] Sun X., Gao H., Yang Y., He M., Wu Y., Song Y., Tong Y., Rao Y. (2019). PROTACs: Great opportunities for academia and industry. Signal Transduct. Target. Ther..

[100] Jude J.A., Panettieri R.A. (2025). IRAK4: Potential therapeutic target for airway disease exacerbations. Trends Pharmacol. Sci..

[101] He M., Cao C., Ni Z., Liu Y., Song P., Hao S., He Y., Sun X., Rao Y. (2022). PROTACs: Great opportunities for academia and industry (an update from 2020 to 2021). Signal Transduct. Target. Ther..

[102] Dragovich P.S. (2022). Degrader-antibody conjugates. Chem. Soc. Rev..

[103] Chan K., Sathyamurthi P.S., Queisser M.A., Mullin M., Shrives H., Coe D.M., Burley G.A. (2023). Antibody-proteolysis targeting chimera conjugate enables selective degradation of receptor-interacting serine/threonine-protein kinase 2 in HER2+ cell lines. Bioconjugate Chem..

[104] Wang L., Ke Y., He Q., Paerhati P., Zhuang W., Yue Y., Liu J., Zhang J., Huang L., Yin Q. (2025). A novel ROR1-targeting antibody-PROTAC conjugate promotes BRD4 degradation for solid tumor treatment. Theranostics.

[105] He S., Gao F., Ma J., Ma H., Dong G., Sheng C. (2021). Aptamer-protac conjugates (apcs) for tumor-specific targeting in breast cancer. Angew. Chem..

[106] Liu Y., Qian X., Ran C., Li L., Fu T., Su D., Xie S., Tan W. (2023). Aptamer-Based Targeted Protein Degradation. ACS Nano.

[107] Zhang G., Yan S., Liu Y., Du Z., Min Q., Qin S. (2025). PROTACs coupled with oligonucleotides to tackle the undruggable. Bioanalysis.

[108] Lv D., Pal P., Liu X., Jia Y., Thummuri D., Zhang P., Hu W., Pei J., Zhang Q., Zhou S. (2021). Development of a BCL-xL and BCL-2 dual degrader with improved anti-leukemic activity. Nat. Commun..

[109] Cheng J., Dong G., Wang W., Sheng C. (2025). Precise Modulation of Protein Degradation by Smart PROTACs. ChemBioChem.

[110] Chen H., Liu J., Kaniskan H.U.M., Wei W., Jin J. (2021). Folate-guided protein degradation by immunomodulatory imide drug-based molecular glues and proteolysis targeting chimeras. J. Med. Chem..

[111] Liu J., Chen H., Kaniskan H.U., Xie L., Chen X., Jin J., Wei W. (2021). TF-PROTACs enable targeted degradation of transcription factors. J. Am. Chem. Soc..

[112] Fan L., Tong W., Wei A., Mu X. (2024). Progress of proteolysis-targeting chimeras (PROTACs) delivery system in tumor treatment. Int. J. Biol. Macromol..

[113] Zografou-Barredo N.A., Hallatt A.J., Goujon-Ricci J., Cano C. (2023). A beginner’s guide to current synthetic linker strategies towards VHL-recruiting PROTACs. Bioorganic Med. Chem..

[114] Hines J., Gough J.D., Corson T.W., Crews C.M. (2013). Posttranslational protein knockdown coupled to receptor tyrosine kinase activation with phosphoPROTACs. Proc. Natl. Acad. Sci. USA.

[115] Uliassi E., Bolognesi M.L., Milelli A. (2025). Targeting Tau Protein with Proximity Inducing Modulators: A New Frontier to Combat Tauopathies. ACS Pharmacol. Transl. Sci..

[116] Zhao C., Dekker F.J. (2022). Novel Design Strategies to Enhance the Efficiency of Proteolysis Targeting Chimeras. ACS Pharmacol. Transl. Sci..

[117] Hu Z., Chen P.-H., Li W., Douglas T., Hines J., Liu Y., Crews C.M. (2023). Targeted dephosphorylation of tau by phosphorylation targeting chimeras (PhosTACs) as a therapeutic modality. J. Am. Chem. Soc..

[118] Hu Z., Chen P.-H., Li W., Krone M., Zheng S., Saarbach J., Velasco I.U., Hines J., Liu Y., Crews C.M. (2024). EGFR targeting PhosTACs as a dual inhibitory approach reveals differential downstream signaling. Sci. Adv..

[119] Negi A., Voisin-Chiret A.S. (2022). Strategies to reduce the on-target platelet toxicity of Bcl-xL inhibitors: PROTACs, SNIPERs and prodrug-based approaches. ChemBioChem.

[120] Negi A., Kesari K.K., Voisin-Chiret A.S. (2022). Light-Activating PROTACs in Cancer: Chemical Design, Challenges, and Applications. Appl. Sci..

[121] Xue G., Wang K., Zhou D., Zhong H., Pan Z. (2019). Light-Induced Protein Degradation with Photocaged PROTACs. J. Am. Chem. Soc..

[122] Verma S., Manna D. (2020). Controlling PROTACs with Light. ChemMedChem.

[123] Yang C., Tripathi R., Wang B. (2023). Click chemistry in the development of PROTACs. RSC Chem. Biol..

[124] Noblejas-López M.d.M., Tébar-García D., López-Rosa R., Alcaraz-Sanabria A., Cristóbal-Cueto P., Pinedo-Serrano A., Rivas-García L., Galán-Moya E.M. (2023). TACkling cancer by targeting selective protein degradation. Pharmaceutics.

[125] Liang J., Wu Y., Lan K., Dong C., Wu S., Li S., Zhou H.-B. (2023). Antiviral PROTACs: Opportunity borne with challenge. Cell Insight.

[126] Piret J., Boivin G. (2021). Pandemics Throughout History. Front. Microbiol..

[127] Brüssow H. (2022). The beginning and ending of a respiratory viral pandemic-lessons from the Spanish flu. Microb. Biotechnol..

[128] Miranda M.N.S., Pingarilho M., Pimentel V., Torneri A., Seabra S.G., Libin P.J.K., Abecasis A.B. (2022). A Tale of Three Recent Pandemics: Influenza, HIV and SARS-CoV-2. Front. Microbiol..

[129] Hatchett R., Moreira M. (2024). We can stop the next pandemic, but only if we act now. Telegraph..

[130] Guedeney N., Cornu M., Schwalen F., Kieffer C., Voisin-Chiret A.S. (2022). PROTAC technology: A new drug design for chemical biology with many challenges in drug discovery. Drug Discov. Today.

[131] Oleinikovas V., Gainza P., Ryckmans T., Fasching B., Thomä N.H. (2024). From Thalidomide to Rational Molecular Glue Design for Targeted Protein Degradation. Annu. Rev. Pharmacol. Toxicol..

[132] Dong G., Ding Y., He S., Sheng C. (2021). Molecular Glues for Targeted Protein Degradation: From Serendipity to Rational Discovery. J. Med. Chem..

[133] Li H., Dong J., Cai M., Xu Z., Cheng X.-D., Qin J.-J. (2021). Protein degradation technology: A strategic paradigm shift in drug discovery. J. Hematol. Oncol..

[134] Gao H., Sun X., Rao Y. (2020). PROTAC Technology: Opportunities and Challenges. ACS Med. Chem. Lett..

[135] Zeng S., Huang W., Zheng X., Zhang Z., Wang J., Shen Z. (2021). Proteolysis targeting chimera (PROTAC) in drug discovery paradigm: Recent progress and future challenges. Eur. J. Med. Chem..

[136] Page M.J., McKenzie J.E., Bossuyt P.M., Boutron I., Hoffmann T.C., Mulrow C.D., Shamseer L., Tetzlaff J.M., Akl E.A., Brennan S.E. (2021). The PRISMA 2020 statement: An updated guideline for reporting systematic reviews. BMJ.

